# Mild Hyperthermia Responsive Liposomes for Enhanced In Vitro and In Vivo Anticancer Efficacy of Doxorubicin against Hepatocellular Carcinoma

**DOI:** 10.3390/pharmaceutics13081310

**Published:** 2021-08-21

**Authors:** Muhammad Abdur Rahim, Asadullah Madni, Nayab Tahir, Nasrullah Jan, Hassan Shah, Safiullah Khan, Riaz Ullah, Ahmed Bari, Muhammad Sohaib Khan

**Affiliations:** 1 Department of Pharmaceutics, Faculty of Pharmacy, The Islamia University of Bahawalpur, Bahawalpur 63100, Pakistan; muhammadabdurrahim88@gmail.com (M.A.R.); nasrullahjan14@gmail.com (N.J.); hasanshah342@gmail.com (H.S.); safiullahkhan856@gmail.com (S.K.); 2 College of Pharmacy, University of Sargodha, Sargodha 40100, Pakistan; nayabtahir132@gmail.com; 3 Department of Pharmacognosy, College of Pharmacy King Saud University, Riyadh 12372, Saudi Arabia; rullah@ksu.edu.sa; 4 Department of Pharmaceutical Chemistry, College of Pharmacy King Saud University, Riyadh 12372, Saudi Arabia; abari@ksu.edu.sa; 5 College of Pharmacy, Dongguk University, Seoul 10326, Korea; muhammadsohaibkhan786@gmail.com

**Keywords:** temperature responsive liposomes, doxorubicin, Korsmeyer–Peppas model, HepG2 cells, MCF-7 cells

## Abstract

The current study is aimed to fabricate doxorubicin (Dox) loaded mild temperature responsive liposomes (MTLs) by thin film hydration technique for enhanced in vitro and in vivo anticancer efficacy against hepatocellular carcinoma. The aforementioned Dox loaded MTLs were developed and optimized with extrusion and drug loading techniques. The optimized MTLs were in optimum size range (118.20 ± 2.81–187.13 ± 4.15 nm), colloidal stability (−13.27 ± 0.04 to −32.34 ± 0.15 mV), and enhanced entrapment of Dox (28.71 ± 2.01–79.24 ± 2.16). Furthermore, the optimized formulation (MTL1-E_(AL)_) embodied improved physicochemical stability deducted by Fourier transform infra-red (FTIR) spectroscopy and mild hyperthermia-based phase transition demonstrated from differential scanning calorimetry (DSC). An in vitro drug release study revealed mild hyperthermia assisted rapid in vitro Dox release from MTLs-E_(AL)_ (T_100%_ ≈ 1 h) by Korsmeyer–Peppas model based Fickian diffusion (*n* < 0.45). Likewise, an in vitro cytotoxicity study and lower IC_50_ values also symbolized mild hyperthermia (40.2 °C) based quick and improved cytotoxicity of MTL1-E_(AL)_ in HepG2 and MCF-7 cells than Dox. The fluorescence microscopy also represented enhanced cellular internalization of MTL1-E_(AL)_ at mild hyperthermia compared to the normothermia (37.2 °C). In addition, an in vivo animal study portrayed the safety, improved anticancer efficacy and healing of hepatocellular carcinoma (HCC) through MTL1-E_(AL)_. In brief, the Dox loaded MTLs could be utilized as safe and effective therapeutic strategy against HCC.

## 1. Introduction

The oncogenesis of malignant tumors is predominantly concerned with rapid abnormal proliferation of cells followed by the invasion into nearby tissues and subsequently metastasized to far tissues and organs [1]. The recently reported data suggest that hepatocellular carcinoma (HCC) is listed among the top five causes of mortalities all around the globe and mostly associated with extensive alcohol consumption, hepatitis B and C viruses and fatty liver [2,3]. The basic treatment protocols associated with variety of cancers include surgery, radiation, and chemotherapy. Among them, surgery and radiotherapy are alarmed with maximal risks as they belong to maximal invasive protocols [4,5]. In contrast, chemotherapy represents an ideal and most convenient treatment strategy for HCC, but the nonspecific localization and toxicity is of great concern regarding the use of chemotherapeutics alone [6].

The nanotechnology based targeted delivery of chemotherapeutic agent(s) revolutionized conventional chemotherapy by providing safe and effective therapeutic strategies against various malignancies [7]. The dawn of liposomes as a drug delivery system (DDS) has been reported in the early 60’s and is involved in continual advancements regarding pharmacokinetics and pharmacodynamics enhancement of therapeutic agents [8]. The liposomes as natural lipid based drug loaded vesicles successfully remained attention seeker for the drug delivery scientists regarding targeted delivery, diagnosis and theranostic applications [9]. The modern research enabled the achievement of novel PEGylated, nanoliposomes (≤200 nm) for stealth effects and tumor specific localization through enhanced permeation and retention effects (EPR) [10]. Among various types of drug delivery systems, thermo-responsive liposomal drug delivery systems are the utmost explored regarding treatment strategies for different types of malignancies. The aforementioned systems are developed by using the combination of appropriate lipids in conjunction with chemotherapeutic agent to achieve the proper vesicular structure and release the drug by varying the temperature. At or below normal body temperature, the lipid mixture used in thermoresponsive liposomes retain the payload of drug, whereas the drug is released at mild hyperthermia associated with local temperature of the tumor mileu [11]. In general, the hyperthermia and chemotherapy both are toxic to normal body cells and cancer cells. In the current study, mild hyperthermia (temperature associated with tumor microenvironment) responsive PEGylated (stealth) liposomes in conjunction with chemotherapeutic agent (Dox) are used to achieve improved and selective targeting, i.e., toxic effects in cancer cells without affecting or harming normal cells [12]. Regarding fabrication of liposomes, in thin film hydration technique, the drug may be trapped within vesicle by active or passive loading technique. The passive loading technique provide better entrapment of lipophilic drug within vesicles upon self-assembling of liposomes. While the hydrophilic drugs like doxorubicin provide limited entrapment as the hydration volume is limited within the core compared to the outer environment. Therefore, the active loading technique provide better trapping of hydrophilic drugs within the vesicles compared to passive loading. In pH gradient based active loading specifically in Myocet (and in the current study) the acidic buffer inside and the basic buffer outside the vesicle established. The aforementioned transmembrane gradient act as a driving force for the loading of Dox inside the vesicles [13].

In addition, the tumor specific targeting through EPR by Dox loaded PEGylated liposomes and thermoresponsive liposomes (TLs) for treatment of various types of cancers successfully achieved FDA approval (Doxil^®^/Caelyx^®^, Myocet^®^, Lipo-Dox^®^, and ThermoDox^®^) [14,15]. Dox is an anthracycline derivative and topoisomerase II intercalating poison for DNA damage, having antineoplastic effects with a short biological half-life of 1–3 h [15]. The ThermoDox^®^ is associated with the Dox loaded PEGylated TLs indicated for breast cancer and HCC [16].

The present study is based on the fabrication and evaluation of stable and heat triggered tumor specific release, cytotoxicity of TLs by EPR and in vivo healing of liver cancer. Furthermore, the current study also highlighted the use of thermoresponsive lipids associated with ThermoDox^®^ and active loading (AL) technique concerned with Myocet^®^. The major drawback regarding ThermoDox^®^ associated with Phase III clinical trials is that it is available in liquid liposomal dispersion form and require strict storage conditions. It requires −60 °C freezing and thawing at room temperature for 1 h before administration, which may decline the marketing acceptance in future [16]. Therefore, in the current study, the features of ThermoDox^®^ and Myocet^®^ have been combined to improve the storage stability and shelf-life of MTLs. In short, the current study presented the achievement of safe, effective and stable Dox loaded nanosized TLs for management of HCC [17].

## 2. Materials and Methods

### 2.1. Materials

Doxorubicin (Dox; molecular weight 579.99 atomic mass units (amu); aqueous solubility of 1.18 mg/mL) and Cholesterol (chol; molecular weight 386.65 amu) were procured from Mesochem, Beijing, China. Diplamitoylphosphatidylcholine (DPPC; molecular weight 734.039 amu) and *N*-(carbonyl-methoxypolyethylene glycol-2000)-1,2-distearoyl-*sn*-glycero-3-phosphoethanolamine (DSPE-(PEG)_2k_; molecular weight 2810 amu) were acquired as generous donations from Lipoid^®^ GmbH, Ludwigshafen am Rhein, Germany. Lyso-Phosphatidylcholine (L-PC; molecular weight 495.63 amu) and diethylnitrosamine (DENA; molecular weight 102.14 amu) were purchased from Avanti polar lipids^®^, Alabaster, AL, USA. Chloroform of analytical grade was purchased from Merck, Kenilworth, NJ, USA and Track etched 100 nm polycarbonate membrane based Mini extruders (Nanosizer Mini^®^) were purchased from T&T scientific corporations, Knoxville, TN, USA. The distilled water was used during experimentation attained from the distillation apparatus (IRMECO^®^, Schwarzenbek, Germany).

### 2.2. Preparation of Dox-Loaded Thermoresponsive Liposomes

The two triads of Dox loaded thermoresponsive liposomes including elevated temperature responsive liposomes (ETLs) and mild temperature responsive liposomes (MTLs) were developed. The aforementioned liposomes were fabricated by thin lipid film hydration technique as suggested by Kim et al., 2014, with slight modifications [18]. In brief, the lipid mixture containing DPPC, DSPE-(PEG)_2k_ and chol for ETLs whereas, L-PC, DPPC, DSPE-(PEG)_2k_, and chol for MTLs equivalent to 200 µmol in total were used, with different micromolar ratios of lipid mixtures as given in Table 1. In addition, the micromolar concentration of DSPE-(PEG)_2k_ was optimized and kept constant such that, the varied micromolar ratios of L-PC, DPPC and chol and fixed molar concentration of DSPE-(PEG)_2k_ was used in the succeeding triad. In both the triads the concentration of Dox was kept constant (4 mg). The lipid mixture was dissolved in chloroform and subsequently subjected to evaporation (Hei-VAP Core Rotavapour®, Heidolph, Schwabach Germany) for 30 min at 37 °C temperature and 90 rpm angular speed to achieve dried lipid film. The overnight vacuum desiccation was done to ensure the removal of traces of solvent if any. Finally, the dried lipid film was subjected to hydration and drug loading through (i) conventional hydration (CH) technique (Phosphate buffer saline (PBS) of pH 7.4; and (ii) active loading (AL) technique (comprehending six formulations each).

Regardless of the technique, in both the cases the Dox was loaded during hydration of the liposomes. In CH with PBS initially Dox-PBS solution was prepared by incorporating 4 mg Dox in 5 mL of PBS and then added to the 5 mL dispersion of lipid film in PBS followed by vigorous stirring.

In the latter case, 5 mL lipid film-ammonium sulphate dispersion was achieved by incorporating lipid film in 120 mM ammonium sulphate buffer followed by vigorous stirring. After that, 4 mg of Dox was dissolved in 145 mM sodium chloride solution, followed by intermixing of both the above-mentioned dispersions with continual vigorous stirring. The completion of the AL was determined through the precipitation of drug loaded TLs [19]. The TLs achieved through the aforementioned technique were sonicated for 15 min (ELMA E-30 H Elmasonic, Singen, Germany) and homogenized (Silent Crusher, Heidolph, Germany) at 15 K rpm for 30 min with or without extrusion through track-etched 100 nm polycarbonate membrane (Nanosizer Mini^®^, T&T scientific corporations, Knoxville, TN, USA). The resultant liposomes dispersion was then stored at 4 °C in the screw capped vials before further evaluation [17].

### 2.3. Analysis of Vesicle Size, PDI, and Zeta Potential

The vesicle size as the precursor of evidence regarding passive tumor targeting (EPR), polydispersity index for the size distribution and Zeta potential for the estimation of colloidal stability of ETLs_(AL)_ and MTLs_(AL)_ were estimated through Malvern instruments (Zetasizer and Zeta Potential analyzer version 7.12, Malvern, UK) by dynamic light scattering (DLS) technique. Initially, all the formulations achieved through AL (six formulations) were selected and categorized into two groups. Before extrusion through track-etched polycarbonate membrane of 100 nm they were considered as, non-extruded elevated temperature responsive liposomes (ETLs-NE_(AL)_) and mild temperature responsive liposomes (MTLs-NE_(AL)_) and after extrusion as, extruded elevated temperature responsive liposomes and mild temperature responsive liposomes (ETLs-E_(AL)_ and MTLs-E_(AL)_). The partition into aforementioned groups was done to evaluate the effect of track-etched 100 nm polycarbonate membrane furnished mini extruders (Nanosizer Mini^®^, T&T scientific corporations, Knoxville, TN, USA) on size, PDI, and zeta potential of the developed formulations [20].

### 2.4. Entrapped Drug Contents (%)

The percent entrapped drug contents (% EDC) of the extruded Dox loaded liposomes ETLs-E (ETL1-E, ETL2-E and ETL3-E) and MTLs-E (MTL1-E, MTL2-E, and MTL3-E) as mentioned before and illustrated in (Table 1) were determined by an indirect method. In brief, initially the thermoresponsive liposomes in which Dox was loaded by conventional hydration {ETLs-E_(CH)_ and MTLs-E_(CH)_} were centrifuged (Sigma-Aldrich, Darmstadt, Germany) at 12 K rpm for 30 min followed by manual withdrawn of supernatant. The process was repeated thrice followed by precise dilution of supernatant with buffer (PBS; pH 7.4) and the contents were estimated through calibration curve by taking absorbance through UV–visible spectrophotometer (IRMECO, U2020, Schwarzenbek, Germany) at 488 nm [21]. The same procedure was repeated for the thermoresponsive liposomes in which Dox was loaded by active loading technique (ETL1-E_(AL)_-ETL3-E_(AL)_and MTL1-E_(AL)_-MTL3-E_(AL)_). However, in this case, the supernatant was initially converted to phosphate buffer saline (PBS, pH 7.4) by incorporating the remaining excipients of PBS [22]. In both the cases the % EDC was calculated through calibration curve by the formula

(1) $$\%\;\mathrm{EDC}=\frac{TDC-UDC}{TDC}\times 100$$

where, % EDC represents “percent entrapped drug contents”, *TDC* represents “Total drug contents” and *UDC* represents “Unentrapped drug contents”.

### 2.5. Morphological Analysis

A small amount of optimized formulation (MTL1-E_(AL)_) was mounted on the membrane of the copper grid as thin layer of liposomes dispersion while excess was removed through the filter paper The sample was made conductive followed by passing the sample through liquid nitrogen and analyzed through transmission electron microscope (JEM-2200FS, JEOL, Akishima, Tokyo, Japan) at 100 KV applied voltage to get images at different magnifications and resolutions [23].

### 2.6. Thermal Analysis

The thermal analysis of Dox, DPPC, physical mixture (PM) containing equimolar ratios of Dox, DPPC, DSPE-(PEG)_2k_, and chol, ETL1-E_(AL)_ and MTL1-E_(AL)_ was performed by DSC (Simultaneous Thermal Analyzer (STA) 6000, Perkin Elmer, CA, USA) to evaluate the hyperthermia based behavior of formulation components and selected formulations. The change in mass or heat flow as a function of temperature in the range of 15 to 400 °C at 10 °C/min was determined for DSC analysis [24].

### 2.7. Physicochemical Stability and Compatibility Analysis

The physicochemical stability analysis of Dox, DPPC, DSPE-(PEG)_2k_, physical mixture (PM) containing equimolar ratios of all the formulation components (Dox, DPPC, DSPE-(PEG)_2k_, and chol) and optimized formulation (MTL1-E_(AL)_) was carried out by attenuated total reflectance (ATR) based Fourier transformed infrared spectroscopy (FTIR). (Bruker, Tensor 27 series, Berlin, Germany). The interferogram was constructed between wavenumber and percent transmittance in the range of 4000–500 cm^−^^1^ and 85–100% respectively. In addition, the chemical structures of the aforementioned components were drawn by ChemDraw 8.0. The modification in peaks or shifting of peaks were considered for the physical/chemical instability and/or incompatibility among formulation components [25].

### 2.8. In Vitro Drug Release Analysis

The in vitro drug release study of ETLs-E_(AL)_ and MTLs-E_(AL)_ was performed by USP Type II apparatus using dialysis tube of 15,000 Da molecular weight cut-off (15 K Da MWCO), 4.09 nm pore size and 40.35% porosity. The temperature was maintained at 37 ± 0.5 °C (normothermia), 40 ± 0.5 °C (mild hyperthermia), and 42 ± 0.5 °C (elevated temperature) and stirring speed was adjusted at 50 rpm [26]. The reason of using the dialysis tube with aforementioned properties may be justified with the reported literature, which states that the pore size should be so small that it can retain the nanoparticulate based drug delivery systems for a variety of biomedical applications. In counter-wise, the pore size should be large enough for the passage of the drug through diffusion. In addition, the volume of the dissolution apparatus should be kept 6–10 times higher than the volume included inside the dialysis membrane [27]. The ETLs-E_(AL)_ and MTLs-E_(AL)_ equivalent to 1 mg of drug was incorporated in 200 mL of PBS (pH 7.4). The separation of aliquots of 5 mL samples from dispersion medium followed by incorporation of an equal volume of fresh medium was established to maintain sink conditions. The obtained samples were filtered and analyzed through UV/Visible spectrophotometer (IRMECO U2020, Germany) at 488 nm. The amount of the drug was estimated by calibration curve followed by the assessment of drug release by plotting cumulative drug release (%) against time (hours) [28].

### 2.9. Statistical Analysis

The one-tailed Student’s *t*-test was used to evaluate statistical difference among various formulation parameters such as vesicle size, entrapped drug contents and in vitro drug release. For the evaluation parameters including zeta size, zeta potential, PDI, % EDC and in vitro drug release data the *n* was kept as *n* = 3. On the contrary, for the in vitro cytotoxicity and in vivo healing study the *n* was kept as *n* = 5. SD and *p* values were calculated where applicable [29].

### 2.10. Mathematical Modeling

The mathematical modeling was applied through DD solver.xla representing an Add-in of Microsoft Excel using different kinetic models including zero order, first order, Higuchi and Korsmeyer–Peppas model. The aforementioned models were applied to determine the mechanism of drug release from Dox loaded ETLs-E_(AL)_ and MTLs-E_(AL)_ at normothermia (37 ± 0.5 °C), mild hyperthermia (40 ± 0.5 °C), and elevated temperature (42 ± 0.5 °C), through regression coefficient (R^2^) and values of release exponent (*n*) [30].

### 2.11. In Vitro Cytotoxicity and Cell Uptake Studies

The in vitro cytotoxicity and cell uptake studies were performed by MTT assay and fluorescence microscopy at normothermia and mild hyperthermia maintaining suitable condition.

#### 2.11.1. Cell Culture

Human hepatocellular carcinoma (HCC/HepG2) and human breast cancer (MCF-7) cell lines were used for the current study. The cells were cultured in Roswell Park Memorial Institute (RPMI) 1640 medium, augmented with 10% fetal bovine serum (FBS), 45 IU/mL penicillin and 45 IU/mL streptomycin. The aforementioned components were facilitated with 95% humidity, and 5% CO_2_ at 37 °C in incubator.

#### 2.11.2. In Vitro Cytotoxicity Study

The cytotoxic evaluation of free Dox, and Dox loaded liposomes was performed through MTT assay. In brief, human HCC (HepG2) and human breast cancer (MCF-7) cell lines were seeded in 96 well plates as 10,000 cells per well for overnight in the medium of Dulbecco’s modified Eagle medium (DMEM) containing 10% FBS, 1% antibody and the pre-mentioned penicillin and streptomycin facilitated with 95% humidity and 5% CO_2_ at 37 °C temperature for attachment. After washing with PBS the cells were treated with free Dox (0–10 µg/mL, *n* = 5) and Dox loaded liposomes (0–10 µg Dox/mL, *n* = 5) at 37.2 °C for 24 h and 48 h. The same procedure was carried out using an additional empty incubator set at 40.2 °C (mild hyperthermia) and when the temperature reached to 40.2 °C the procedure was carried out for 1 h. The cells were washed with PBS twice to remove the residual drugs and then, the 20 µL MTT solution (5 mg/mL) was added in each well and incubated for 3–4 h at 37 °C until precipitated in formazan. The formazan was dissolved in suitable vehicle and absorbance was measured at 570 nm using the microplate reader (Greiner 96 Flat Bottom Transparent Polystyrol, Sigma, St. Louis, MO, USA). The Dox solution and hyperthermia were considered as controls. In addition, the maximal-half inhibitory concentration (IC_50_) values of Dox solution and MTL1-E_(AL)_ at normothermia (37.2 °C) after 24 h and 48 h and mild hyperthermia (40.2 °C) after 1 h were also calculated.

#### 2.11.3. Fluorescence Microscopy Based Cell Uptake Study

After completion of cytotoxic assay, the fluorescence-based cell uptake was detected through fluorescence microscopy at image station of University of Lahore by Confocal Laser Scanning microscope (Leica TCS SP5 Inverted Supercontinuum X, Leica Microsystems, Chicago, IL, USA). Initially the HepG2 cells were seeded in multi-chambered (eight chambered) glass bottom dishes containing 10,000 cells per dish at 37 °C for 24 h and then the growth media was removed and the cells were washed with Hank’s balanced salt solution-HEPES (HBSS-HEPES, pH 7.4) buffer. After that Dox solution and Dox loaded liposomes using 10 µg Dox/mL concentration were incorporated in each dish and incubated at 37 °C for preselected time spans. The RPMI media was considered as negative control. The cells were washed for multiple times to remove free nanoparticles and then the nuclei staining of HepG2 cells was done by incubating the cells with DAPI blue for 5 min at 25 °C. The extra amount of stain was removed by multiple washings with PBS and then the cells were fixed by using the formaldehyde solution. The samples were visualized under confocal laser scanning microscope.

The aforementioned procedure was repeated for the above-mentioned samples at mild hyperthermia (40.2 °C) through empty incubator for 1 h as well (as discussed earlier). The aforementioned samples were then observed through fluorescence microscope.

### 2.12. In Vivo Cancer Induction and Healing Study

The in vivo study was carried out using albino mice in accordance with Organization for Economic Co-operation and Development (OECD) guidelines. The in vivo study was performed in the animal house of the Islamia University of Bahawalpur after written consent achieved from Pharmacy animals and ethics committee (PAEC; PAEC number is 20/31/PAEC). Total 30 albino mice with the mean body weight of 29.6 g were selected for the study, and were divided into three groups as; control group (A), intoxicated group (B), and healing/healed group (C) as suggested by Fatimah and Omaima, 2016 [31]. Initially, all the mice were divided into two groups i.e., control group (A) with 10 mice and intoxicated group (B) having 20 mice. The proper temperature (25 °C) and hygiene was maintained throughout the experiment and the food and water was provided ad libitum. The intoxication and healing of the mice were depicted through the movements of mice.

Initially, 1% diethylnitrosamine (DENA) *w/v* solution was prepared by incorporating 99 mL of water for injection in the vial containing 1 g/mL of DENA. The DENA equivalent to 83 mg/Kg was administered to the intoxicated group for six weeks as once weekly dosing through intraperitoneal route to induce HCC. On the contrary, the control group was injected water for injection for six weeks through intraperitoneal route. Eight mice died in the intoxicated group during the dosing of DENA for six weeks and the remaining 12 were further divided into two groups out of which six were dissected and indorsed for histopathological studies. The remaining six mice were initially provided food and water ad libitum for one week and then subjected for healing (Group C) through vesicle-based chemotherapy. The optimized formulation (MTL1-E_(AL)_) equivalent to 5 mg/Kg of Dox was administered to six mice through intraperitoneal injections as three doses weekly for three weeks (8th, 9th, and 10th week). Regarding an in vivo study, the selection of amounts of cancer inducing agent (83 mg/kg of body weight) and the equivalent amounts of Dox (5 mg/kg of body weight) in the optimized formulation (MTL1-E(AL)) was based on the already reported study [32]. One mice died during the dosing of the formulation, and the remaining five mice were also dissected at the mid of 11th week and subjected for histopathological study (*n* = 5) by the protocols suggested by Pittala et al., 2018 [33].

## 3. Results and Discussion

### 3.1. Preparation of Dox-Loaded Thermoresponsive Liposomes

Total twelve liposomes formulations were developed using the lipids mixture used in ThermoDox^®^ followed by hydration of thin lipid film similar to Myocet^®^. By combining the features of ThermoDox^®^ and Myocet^®^ it could be considered in future as the reconstitution of the lipid film followed by utilization of mild hyperthermia source for the treatment of cancer. As strict storage conditions are required for ThermoDox^®^ which may decline its marketing acceptance, therefore, the features of ThermoDox^®^ as well as Myocet^®^ have been combined in the current study. The above mentioned procedure is the prerequisite for stable formulations as dried lipid mixtures are more stable and with improved shelf-life than liquid preparations, as reported earlier and recently, by our research group [34,35]. The aforementioned formulations were primarily partitioned into two batches with six formulations each. Each batch of six formulations was divided into two sets containing couple of triads in each set. The first batch of formulations represented the achievement of hydration of the lipid mixture and conversion into liposomes through loading of Dox by CH—i.e., one triad of ETLs_(CH)_ (ETL1_(CH)_ to ETL3_(CH)_) and one triad of MTLs_(CH)_ (MTL1_(CH)_ to MTL3_(CH)_). Likewise, another batch of six formulations was composed of one triad of elevated temperature responsive liposomes (ETL1_(AL)_-ETL3_(AL)_) and one triad of mild temperature responsive liposomes (MTL1_(AL)_-MTL3_(AL)_) associated with Dox loading by AL. In AL using ammonium sulphate (NH_4_SO_4_) and sodium chloride (NaCl) based pH gradient technique, the ammonia (–NH_3_) from NH_4_SO_4_ moves outside the vesicles leaving a proton (H+) inside the vesicles and the pH gradient develops. After escaping from the vesicles, the amine group (–NH_2_) of –NH_3_ interacts with Dox and converts into Dox-NH_2_. The resultant Dox-NH_2_ then moves from outside into the inside of the vesicles as reported by Abraham et al., 2005 [19].

The AL based Dox loaded liposomes formulations were selected for the study and evaluated for variety of formulation parameters before extrusion as non-extruded temperature responsive liposomes (ETL1-NE_(AL)_-ETL3-NE_(AL)_ and MTL1-NE_(AL)_-MTL3-NE_(AL)_) and after extrusion as extruded temperature responsive liposomes (ETL1-E_(AL)_-ETL3-E_(AL)_ and MTL1-E_(AL)_-MTL3-E_(AL)_). The varied micromolar concentrations of DPPC, DSPE-(PEG)_2k_ and chol were used in the first triad, and varied micromolar concentrations of L-PC, DPPC, DSPE-(PEG)_2k_, and chol were used in the succeeding triad (total 200 µmol) as tabulated in Table 1. In both the aforementioned triads fixed concentration of Dox (4 mg) was used.

It has been reported by Kneidl et al., 2014, that, DPPC is almost complementary for achieving phase transition around 42–43 °C. Furthermore by incorporating Lysolipid (L-PC), and keeping DPPC ≥ 90% in micromoles, phase transition occur at lower temperature (39–41 °C) and provides rapid drug release at lower temperature (39–41 °C) [36]. In the current study two different triads (ETLs-E_(AL)_ and MTLs-E_(AL)_) based on the phase transition temperature were developed and similar lipid composition was used and so similar results were obtained (as discussed later). The ETLs-E_(AL)_ are developed with the eutectic mixtures of lipid which remain stable at normal body temperature (37 °C), but become unstable at its phase transition temperature (42 °C). Likewise, the MTLs-E_(AL)_ remain stable at normal body temperature (37 °C), but become unstable at its phase transition temperature (40 °C) by incorporating L-PC. The current behavior also substantiated the retention of payload at body temperature and release of drug at cancer site.

In addition, it has also been reported by Abraham et al., 2005, that to protect increase in phase transition temperature, the amount of cholesterol should be less than 30% of the total lipid mixture. Moreover, Abraham et al., 2005, also reported that for maximum entrapment of Dox within vesicular bilayer the Dox should be less than 30% of the total lipid mixture weight [19]. Therefore, in the current study, the cholesterol and Dox contents are kept minimum.

All the formulations were characterized through a variety of formulation parameters which have been discussed below.

### 3.2. Analysis of Vesicle Size, PDI, and Zeta Potential

The effect of extrusion (Nanosizer Mini^®^, USA) on the vesicle size, PDI and Zeta potential of the developed Dox loaded (through AL) thermoresponsive liposomes formulation was estimated. The size range of the ETLs-E_(AL)_ was found from 150.4 ± 2.3 nm to 500.8 ± 9.9 nm, whereas the size of MTLs-E_(AL)_ was in the range of 318.5 ± 2.9 nm–345.4 ± 3.0 nm. The vesicle size of all the non-extruded formulations demonstrated the large vesicle size—i.e., above 200 nm which is not acceptable for passive tumor targeting through EPR effect. On the contrary, the extruded ETLs_(AL)_ and MTLs_(AL)_ were found in the acceptable size ranges of 118.2 ± 2.81 to 187.1 ± 4.15 nm and 144.6 ± 2.43 to 172.7 ± 3.15 nm as illustrated in Table 1 and Figure S1 (Supplementary file). In short, the TLs-E_(AL)_ were found in the optimum size range required for tumor targeting through EPR (<200 nm) and well in accordance with the reported literature [37].

Moreover, in both the sets, it was found that concentration of DSPE-(PEG)_2k_ and chol has a direct relation with the vesicle size. Similar behavior was also reported by Madni et al., 2018 and Hsu and Chen, 2017 [34,38]. The micromolar concentration of DSPE-(PEG)_2k_ was optimized and kept constant for the next triad as tabulated in Table 1. The varied micromolar ratios of L-PC, DPPC, and chol keeping the fixed molar concentration of DSPE-(PEG)_2k_ were used in the succeeding triad. The PDI of the developed formulations were also influenced by extrusion (Nanosizer Mini^®^, USA), as the extruded formulations showed better monodispersity in both the cases of ETLs_(AL)_ (0.25 ± 0.02 to 0.43 ± 0.01) and MTLs_(AL)_ (0.21 ± 0.02 to 0.29 ± 0.01) compared to the non-extruded ETLs_(AL)_ (0.41 ± 0.01 to 0.64 ± 0.04) and MTLs_(AL)_ (0.28 ± 0.01 to 0.79 ± 0.03) formulations as represented in Table 1 and Figure S1 (Supplementary Materials). Furthermore, the statistical analysis represented the significant effect of extrusion on the achievement of nanosized liposomes and monodispersity (*p* < 0.05) of liposomes which is also in accordance with the data reported by Ong et al., 2016 [39]. The Zeta potential values of the ETLs-NE_(AL)_ and ETLs-E_(AL)_ were found in the range of −13.08 ± 0.12 to −22.52 ± 0.14 mV and −13.27 ± 0.04 to −22.63 ± 0.31 mV. Likewise, Zeta potential values of the MTLs-NE_(AL)_ and MTLs-E_(AL)_ were found in the range of −28.22 ± 0.39 to −31.69 ± 0.13 mV and −29.32 ± 0.41 mV to −32.34 ± 0.15 mV respectively, as tabulated in Table 1. The achieved Zeta potential depicted the optimum colloidal stability and accordance with the reported literature [40]. In addition, the Zeta potential was not influenced significantly by extrusion (*p* > 0.05), but an increase in the percent micromolar concentration of chol, the zeta potential values also increased. The aforementioned prerogative could be justified with the concept regarding direct link of the cholesterol with the colloidal stability as reported by Madni et al., 2018 [34].

### 3.3. Entrapped Drug Contents (%)

In this study, the %EDC were primarily testified with the loading procedures through which the formulations were achieved. The formulations achieved through AL showed significant improvement (*p* < 0.05) in entrapment of drug contents within the vesicles (29–79%) compared to the formulations achieved through CH (22–52%) as illustrated in Table 1. The reason may be attributed to the reported concept of pH gradient which facilitated the improved drug entrapment within the vesicles [13]. In addition the highest entrapped drug content was found in MTL1-E_(AL)_ which may be verified with the concept reported by Bhatia et al., 2018, regarding effect of phase transition temperature of lipid mixture on entrapment of drug within vesicles [41]. Bhatia et al., 2018 reported that the entrapment of hydrophilic drugs within liposomes are based on the optimum phase transition temperature of the lipids which is 40 °C. Similar phase transition (mentioned below), and percent drug entrapment (Table 1) were also associated with MTL1-E_(AL)_ [42]. The least vesicle size, monodispersity, optimum colloidal stability and enhanced entrapment of Dox was achieved through MTL1-E_(AL)_ and so selected as optimized formulation.

### 3.4. Morphological Analysis

**Figure 1:**
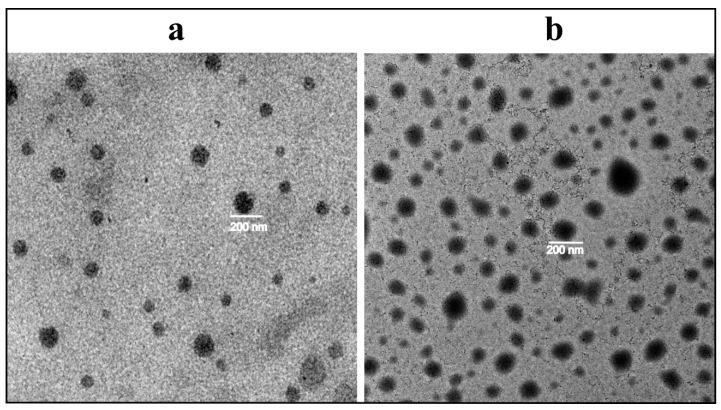
Morphological analysis of ETL1-E_(AL)_ (**a**) and MTL1-E_(AL)_ (**b**) by transmission electron microscope.

The morphological analysis of selected formulations (ETL1-E_(AL)_, MTL1-E_(AL)_) after extrusion was carried out through the TEM. The thermoresponsive liposomes associated with both ETL1-E_(AL)_ and MTL1-E_(AL)_ were found vesicular or hexagonal (Figure 1a,b) in shape. The aforementioned morphology may be associated with active loading of Dox by sulphate gradient (pH gradient) technique and presence of ethanolamines (DSPE-(PEG)_2k_) as reported by Shi et al., 2019 [43]. Almost, all the extruded TLs associated with MTLs-E_(AL)_ and ETLs-E_(AL)_ were of similar vesicle size (≤200 nm) and symmetry, but the vesicles associated with ETL1-E_(AL)_ are smaller. Similar behavior was also found in size analysis through DLS technique. The aforementioned observation collectively provide an evidence regarding monodispersity and efficiency of the development technique. Moreover, the observed morphology is in accordance with the data reported by Chiu et al., 2005 and Shi et al., 2019 [42,43].

### 3.5. Thermal Analysis

**Figure 2:**
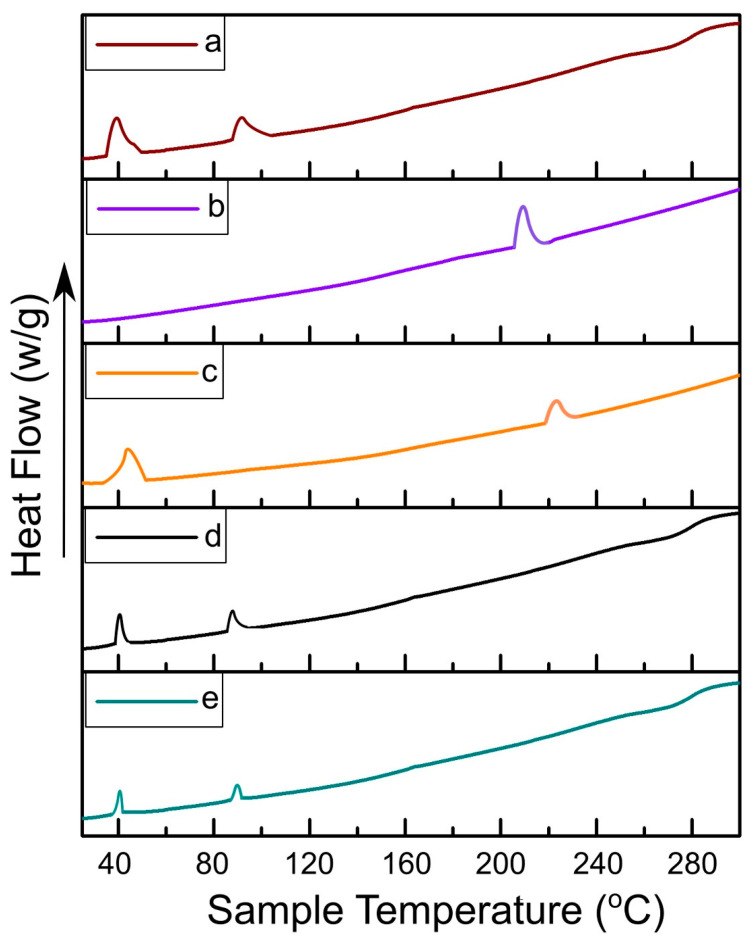
DSC thermograms of DPPC (**a**), Dox (**b**), physical mixture (**c**), ETL1-E_(AL)_ (**d**), and MTL1-E_(AL)_ (**e**).

The thermograms of DSC analysis associated with DPPC, Dox, physical mixture containing equimolar ratios of all the formulation components, ETL1-E_(AL)_ and MTL1-E_(AL)_, were constructed between sample temperature versus heat flow (Figure 2). Based on DSC thermograms DPPC showed endothermic peaks associated with phase transition (T_pt_) at 41.1 °C and fusion or melting endothermic (T_m_) peak at 85 °C, whereas, the Dox represented a single endothermic peak associated with T_m_ at 220.6 °C which is in accordance with the reported literature [44]. The endothermic peaks of the physical mixture were observed distinctly above 42.3 °C and 220.6 °C associated with T_pt_ of DPPC and T_m_ of Dox with slight shifting due to the presence of multiple formulation components. On the contrary, the endothermic peak associated with T_pt_ of the ETL1-E_(AL)_ was observed at 42.3 °C whereas, the T_pt_ of the MTL1-E_(AL)_ was observed exactly at 40.0 °C. The endothermic peak of MTL1-E_(AL)_ associated with T_pt_ at a lower temperature than physical mixture and DPPC clearly depicted the achievement of LCST. Similar behavior was also observed in the in vitro drug release study, as maximal in vitro drug release by ETL1-E_(AL)_ was observed at 42 °C, whereas MTL1-E_(AL)_ displayed maximum drug release at 40 °C (as discussed later). In brief, the achieved results suggested the successful achievement of Dox loaded mild hyperthermia responsive liposomes and accordance with the reported literature [23]. In addition, according to the concept reported by Kneidl et al., 2014, if the hydrophilic drug is entrapped within liposomes then the drug is released at the phase transition temperature of vesicles. The reason is attributed to the concept that the lipid bilayer remains stable at normal body temperature and face instability at phase transition temperature. The instability occur due to the conversion of solid gel phase of lipid mixture to the liquid crystalline phase. In addition, it has also been reported that phase transition around 42–43 °C could be achieved through DPPC based thermoresponsive liposomes. Furthermore, by incorporating lysolipid (L-PC), and keeping DPPC ≥ 90% in micromoles, phase transition occur at lower temperature (39–41 °C). Similar behavior and phase transition temperature has also been achieved in the present study (39–41 °C) [36].

### 3.6. Physicochemical Stability and Compatibility Analysis

**Figure 3:**
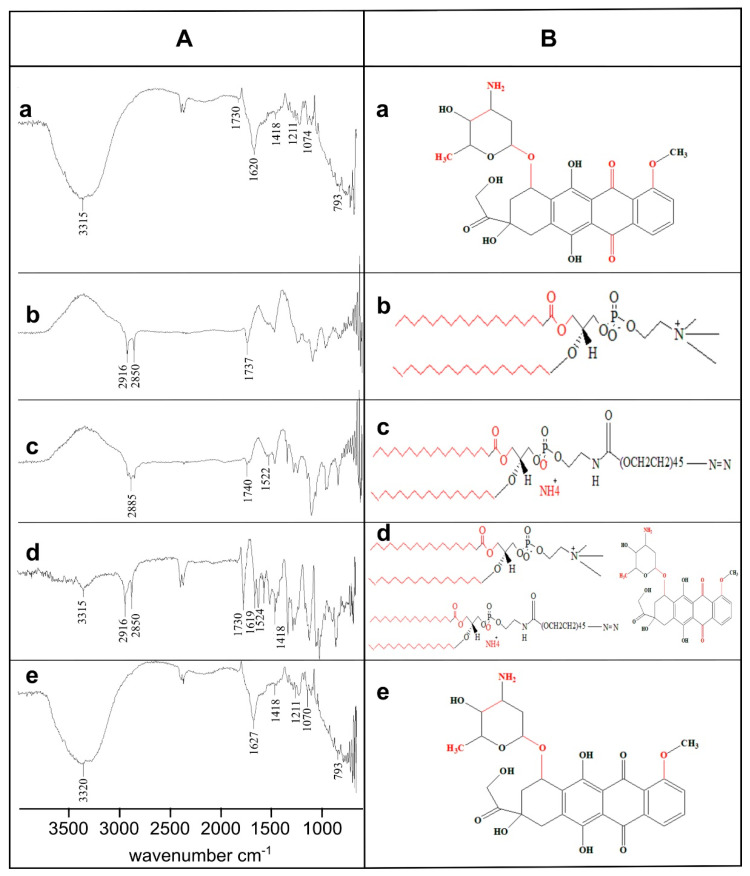
Physicochemical stability and compatibility analysis by FTIR interferogram (**A**) and chemical structure (**B**) of Dox (a), DPPC (b), DSPE-(PEG)_2k_ (c), Physical Mixture (d) and MTL1-E_(AL)_ (e). The observed functional groups have been highlighted with FTIR peaks in “**A**” and as “red highlighted regions” in “**B**”.

The physicochemical stability and compatibility analysis of the Dox, DPPC, DSPE-(PEG)_2k_, physical mixture (PM), and optimized formulation (MTL1-E_(AL)_) was carried out by ATR-FTIR and various functional groups were observed. The Dox represented seven major characteristic peaks associated with FTIR interferogram including 3315 cm^−^^1^, 1730 cm^−^^1^, 1620 cm^−^^1^, 1418 cm^−^^1^,1211 cm^−^^1^, 1074 cm^−^^1^, and 793 cm^−^^1^ (Figure 3A). The aforementioned FTIR peaks depicted the –NH stretching of aliphatic primary amine, –C=O stretching of the aldehyde group, –NH stretching of the amide group I and II, –C–O–C stretching of the ether group, and –C–O stretching of the vinyl ether (highlighted red color regions of Figure 3B). Similar peaks associated with Dox have also been reported by Tahir et al., 2017 and Danmaigoro et al., 2017 [45,46]. Likewise, the DPPC displayed the FTIR peaks at 2916 cm^−^^1^, 2850 cm^−^^1^, and 1737 cm^−^^1^ associated with the –OH stretching of carboxyl group, –CH stretching of alkane and –C=O stretching of the ester group. DSPE-(PEG)_2k_ represented peaks of interferogram at 2885 cm^−^^1^, 1740 cm^−^^1^, and 1522 cm^−^^1^ linked with –CH stretching of alkane, –C=O stretching of ester group and N–O stretching (Figure 3A,B) respectively.

The physical mixture represented four FTIR peaks of Dox at 3315 cm^−^^1^, 1730 cm^−^^1^, 1619 cm^−^^1^, and 1418 cm^−^^1^, two peaks at 2916 cm^−^^1^ and 2850 cm^−^^1^ associated with DPPC and a peak at 1524 cm^−^^1^ related to DSPE-(PEG)_2k_ respectively. Likewise, the formulations represented FTIR peaks at 1418 cm^−^^1^, 1211 cm^−^^1^, and 793 cm^−^^1^ as exact Dox peaks and at 3320 cm^−^^1^ (3315 cm^−^^1^ of Dox), 1627 cm^−^^1^ (1619 cm^−^^1^ of Dox), and 1070 cm^−^^1^ (1074 cm^−^^1^ of dox) associated with slight shifting with respect to the Dox pure drug. The presence of characteristic FTIR peaks of Dox in the formulations depicted the physicochemical stability of the developed formulations and compatibility among formulation components. Furthermore, the slight shifting in FTIR peaks provide sufficient evidence regarding the successful entrapment of Dox in thermoresponsive liposomes [47].

### 3.7. In Vitro Drug Release Analysis

**Figure 4:**
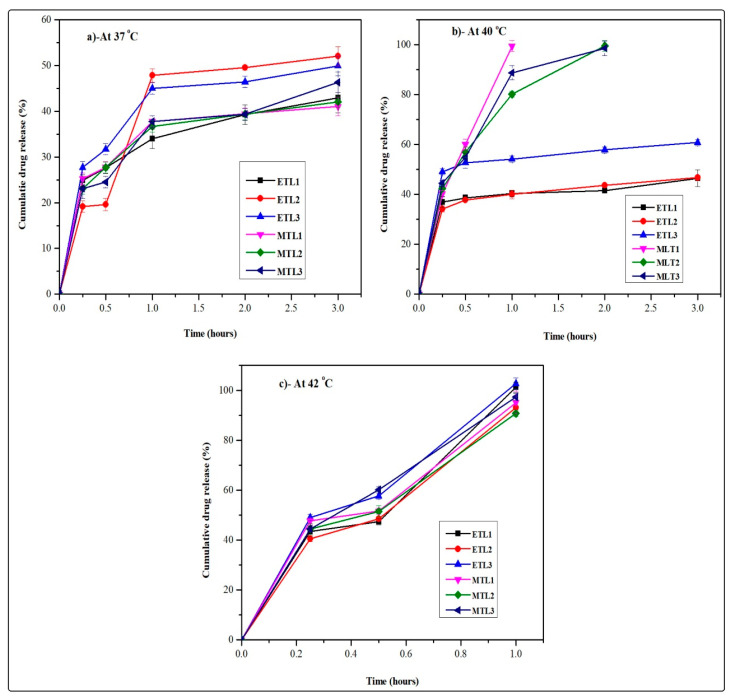
Hyperthermia assisted in vitro drug release profiles of ETLs-E_(AL)_ (ETL1-E_(AL)_-ETL3-E_(AL)_) and MTLs-E_(AL)_ (MTL1-E_(AL)_-MTL3-E_(AL)_) at 37 °C (**a**), 40 °C (**b**), and 42 °C (**c**).

The developed formulations achieved through AL followed by extrusion showed hyperthermia assisted drug release as shown in Figure 4. In brief, at 42 °C (elevated temperature) ETL3-E_(AL)_ showed maximum drug release (≈100%), but in general, all the formulations showed similar behavior and released the drug in 1 h at the aforementioned temperature. The MTLs-E_(AL)_ displayed maximum drug release (≈100%) at 40 °C (mild hyperthermia) among which MTL1-E_(AL)_ represented uppermost and quickest drug release (≈100% in 1 h). The remaining MTLs-E_(AL)_ (MTL2-E_(AL)_ and MTL3-E_(AL)_) also showed maximum drug release at mild hyperthermia but slightly slower and delayed than MTL1-E_(AL)_ (≈100% in 2 h). In contrast, at mild hyperthermia the Dox release from ETLs-E_(AL)_ was found in the range of 46.5–60.8% as shown in Figure 4. In addition, about 50% of the drug release was observed in first hour at 37 °C as well as 42 °C regardless of the change in temperature. The observed behavior associated with initial quick release at 37 °C might be attributed to the release of drug adsorbed on the surface of vesicles or un-encapsulated drug and the initial diffusion of the drug from the liposomes when came in contact with the large amounts of dissolution media. It could be seen that, the higher percent cumulative in vitro drug release was observed at 37 °C in case of ETLs-E_(AL)_ (up to 48%) than MTLs (up to 36%), due to the decreased amounts of encapsulated drug (higher amounts of un-encapsulated drug) in ETLs (up to 53%) compared to MTLs-E_(AL)_ (up to 80%). Likewise, at 42 °C the combined effect of the release of un-encapsulated drug and the instability of the vesicular bilayer was observed. The observed behavior could also provide a perspective regarding an in vivo behavior of the developed Dox-loaded thermoresponsive liposomes, when they come in contact with the biological fluids based on the LADMER concept. These results collectively suggested that the MTLs-E_(AL)_ successfully achieved the LCST and mild hyperthermia assisted Dox release by addition of L-PC to the ETLs_(AL)_. The aforementioned results also predicts an evidence regarding tumor based drug release in externally heated tumors which is in accordance with the reported literature as well [48]. Based on the reported concept by Kneidl et al., 2014, the release of hydrophilic drug entrapped within liposomes occur at the phase transition temperature of vesicles. The reason is attributed to the concept that the lipid bilayer remains stable at normal body temperature and face instability at phase transition temperature. The instability of lipid bilayer occur through conversion of solid gel phase of lipid mixture to the liquid crystalline phase. When this variation occur, the permeability of vesicular bilayer increases, which in turn, facilitates the movement of water from outside to inside and escape of the drug from inside to outside [36].

Similar behavior was also observed in the current study and ETLs-E_(AL)_ presented quick and enhanced drug release at its phase transition temperature (42 °C, depicted from DSC analysis). Likewise, the MTLs-E_(AL)_ portrayed the enhanced and quick release of payload at 40 °C associated with phase transition temperature of MTLs-E_(AL)_ (depicted from DSC analysis). The current behavior also substantiated the retention of payload at body temperature and release of drug at cancer site.

### 3.8. Mathematical Modeling

Based on mathematical modeling, all formulations showed well-fitting in Korsmeyer–Peppas model associated with higher R^2^ values compared to zero order, first order and Higuchi model. In addition, the release exponent value depicted the Fickian diffusion-based mechanism of drug release as the value of *n* was less than 0.45 as given in Table 2. The achieved results are in accordance with the literature reported by Affram et al., 2017 [49], who revealed that the drug release from thermoresponsive liposomes usually follow diffusion based mechanism of drug release and well-fitting in Korsmeyer–Peppas model.

### 3.9. In Vitro Cytotoxicity Study

**Figure 5:**
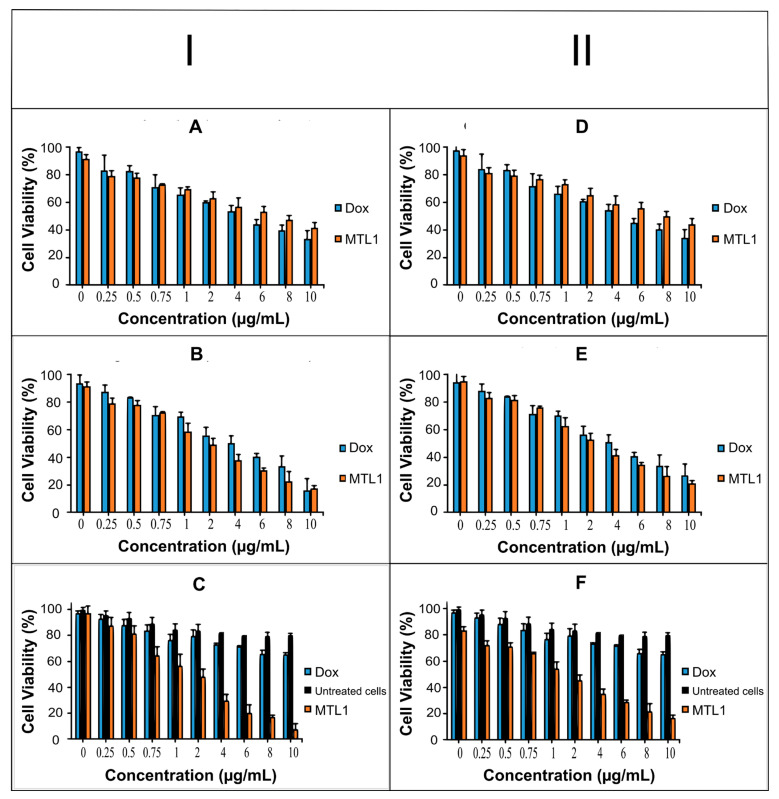
Cell viability evaluation of Dox and Dox loaded liposomes using different concentrations (0–10 µg/mL) after incubation at 37.2 °C for 24 h (**A**) and 48 h (**B**) and cell viability evaluation of Dox, untreated cells and Dox loaded liposomes after incubation at 40.2 °C for 1 h (**C**) using HepG2 (**I**) cell line. Likewise, Cell viability evaluation of Dox and Dox loaded liposomes using different concentrations (0–10 µg/mL) after incubation at 37.2 °C for 24 h (**D**) and 48 h (**E**) and cell viability evaluation of Dox, untreated cells and Dox loaded liposomes after incubation at 40.2 °C for 1 h (**F**) using MCF-7 cell line (**II**).

Based on the MTT assay, the Dox solution represented improved cytotoxicity of HepG2 cells at 37.2 °C (normothermia) after 24 h exposure compared to the MTL1-E_(AL)_ at 10 µg/mL as represented in Figure 5A (I). However, after carrying out the cytotoxicity study for 48 h Dox solution and MTL1-E_(AL)_ both displayed equivalent cytotoxicity and up to 80% of cell killing was achieved at the concentration of 10 µg/mL (Figure 5B). The untreated cells were not observed for cytotoxicity at 37.2 °C (normothermia) as this temperature is not harmful for cells. At 40.2 °C (mild hyperthermia) least/non-significant cytotoxicity to untreated cells was observed. Whereas, the MTL1-E_(AL)_ showed maximum and improved cytotoxicity at 40.2 °C (Figure 5C) after exposure for 1 h compared to Dox solution. The procedure was limited to 1 h because hyperthermia itself causes the temperature and time dependent cytotoxicity of the cells, so the least possible elevation in temperature which is safe for the cells for the minimal possible time spans were used.

The MTT assay based cytotoxic assay at normothermia (37.2 °C) for 24 h (Figure 5D) and 48 h (Figure 5E) and at mild hyperthermia (40.2 °C) for 1 h (Figure 5F) in MCF-7 cells also presented similar behavior, but less cytotoxicity was observed in MCF-7 cells compared to HepG2 cells. In addition, regardless of aforementioned cancer cell lines, MTL1-E_(AL)_ exhibited the enhanced cytotoxicity in MCF-7 cells as well as HepG2 cells. The reason may be attributed to the fact that, ThermoDox^®^ is indicated for breast cancer as well as HCC. In brief the Dox loaded mild hyperthermia responsive PEGylated liposomes could be utilized for targeted delivery and enhanced cytotoxicity in both HepG2 cells and MCF-7 cells and could efficiently be used as chemotherapy-based treatment strategy for HCC.

Moreover, the IC_50_ of MTL1-E_(AL)_ was less than Dox solution at mild hyperthermia (40.2 °C) after 1 h. After 48 h slight better IC_50_ values were observed in case of MTL1-E _(AL)_ than Dox solution at normothermia (37.2 °C) as illustrated in Table 3. Similar behavior was also observed in MCF-7 cells and available literature [50]. In short, the mild hyperthermia (40.2 °C) favor the quick release of Dox and enhanced tumor specific cytotoxicity of HepG2 and MCF-7 cells through MTLs-E_(AL)_ formulations. Additionally, at normothermia (37.2 °C) the MTL1-E _(AL)_ displayed a prolonged release of drug with a slight improvement in cytotoxicity, but at mild hyperthermia (40.2 °C), the MTL1_(AL)_ presented the rapid release of a drug with several folds (>4 folds) enhanced cytotoxicity than Dox solution.

### 3.10. Fluorescence Microscopy Based Cell Uptake Studies

**Figure 6:**
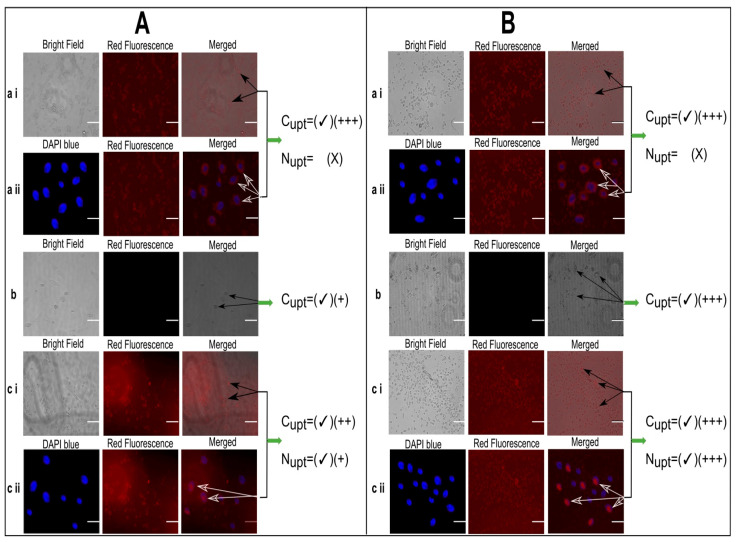
Fluorescence microscopy based cell uptake evaluation of Dox solution (10 µg/mL) (**a**), unloaded liposomes (**b**) and MTL1-E_(AL)_ equivalent to 10 µg/mL Dox concentration (**c**) after incubation for 24 h at 37.2 °C (**A**) and 40.2 °C for 1 h (**B**). (C_upt_ represents cellular uptake, N_upt_ represents nuclear uptake and number of “plus” signs represents the magnitude of cellular uptake. Scale bar is equivalent to 20 µm).

The concentration equivalent to 10 µg/mL of Dox was optimized from the in vitro cytotoxicity study and so kept constant and selected for Fluorescence microscopy-based cell uptake studies. Based on the current study, the fluorescence microscopy (image station^®^) represented the uptake of Dox by HepG2 cells at normothermia (37.2 °C) as well as mild hyperthermia (40.2 °C) after 48 h (Figure 6A,B which portrayed the insignificant effect of temperature on cell uptake of Dox solution by HepG2 cells. In counter wise, the cells have taken up the Dox, but the Dox did not reach the nucleus rather than it was limited to the cell membrane of HepG2 cells. Therefore, instead of presenting the bright labeling within the nucleus, it was observed around the nuclei in Figure 6A (a ii) and Figure 6B (a ii). In addition, the temperature dependent improved cellular uptake of empty liposomes and Dox loaded liposomes (MTL1-E_(AL)_) was observed in Figure 6B (b) and (c i) through bright field microscopy. On the contrary, at normothermia (37.2 °C) limited cell uptake of the vesicles associated with the optimized formulation (MTL1-E_(AL)_) was achieved (Figure 6A (c ii)) after 24 h. In divergence, MTL1-E_(AL)_ displayed the enhanced cell uptake of Dox loaded vesicles incubated at mild hyperthermia (40.2 °C) that could be observed in Figure 6B (c ii). In addition, the Dox was not limited only to the cell membrane in the case of Dox loaded thermosensitive liposomes (ETL1-E_(AL)_, MTL1-E_(AL)_), rather than reached to the nuclei as well. The reason may be attributed with the concept discussed earlier, that the instability of vesicles occur at phase transition temperature which, in turn, causes the leakage and release of the drug from the vesicles. Furthermore, the temperature and time dependent cell uptake of MTL1-E_(AL)_ was observed which is well in accordance with the concept reported by Huff et al., 2007, according to which a slight increase in temperature increased cell uptake of nanoparticles is established and vice versa [51]. The mild hyperthermia based improved cell uptake conceptualized the improved cell uptake of MTL1-E_(AL)_ by cancer cells compared to the normal cells as normal cells are associated with normothermia whereas, the cancer cells and solid tumors are associated with mild hyperthermia. In brief, the aforementioned results revealed the neoplasia specific targeted delivery of the developed active loading based Dox loaded mild temperature responsive liposomes (MTL1-E_(AL)_) against HCC.

### 3.11. In Vivo Cancer Induction and Healing Study

**Figure 7:**
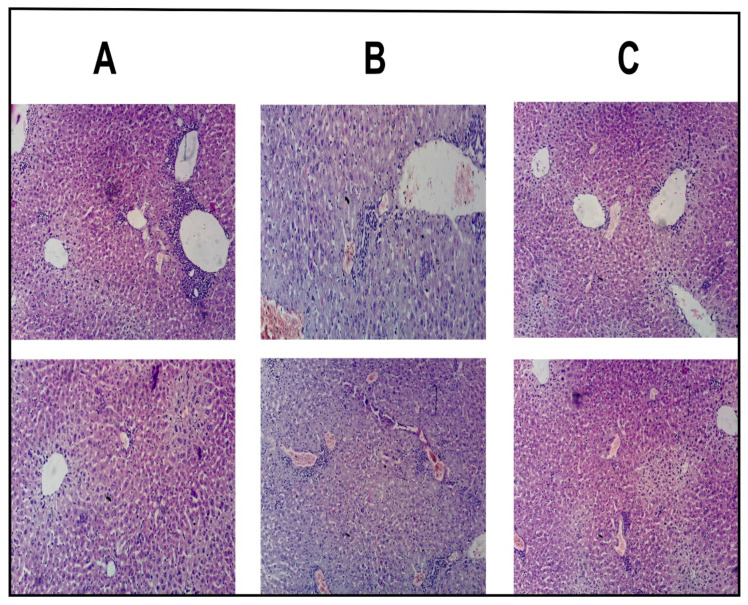
Histopathological evaluation of hepatic cells of albino mice associated with control group (**A**), intoxicated group (**B**), and healed group (**C**).

The physical observation represented that the mice in control group (Group-A) were very energetic and quick regarding movements with increased food and water intake and body weights (mean body weight was 32.7 g). Correspondingly, the histopathological study displayed the lack of any signs of disruption, ulceration and disease state in hepatic cells of control group (Group-A) as illustrated in Figure 7A. In contrast, the physical observation of the mice in intoxicated group displayed minimal motility, lethargic behavior, less food and water intake, and loss in weight (mean body weight was 23.1 g). Based on the aforementioned signs, the mice were roughly guessed for the induction of HCC. The histopathological study of the intoxicated group also verified that, DENA effectively induced HCC as increased proliferation of abnormal hepatic cells could be seen in the Figure 7B. In divergence to the Group-B, the movement, food and water intake and weight of the mice in healed group (Group-C) improved at the end of 10th week (mean body weight was 28.4 g). In addition, the histopathological evaluation of the healed group (Figure 7C) presented the maximum amount of normal cells which might be attributed with the green signal regarding efficient treatment and healing of HCC through MTL1-E_(AL)_. The achieved results are well in accordance with the reported literature as well [32,52].

## 4. Conclusions

Temperature responsive liposomes were successfully fabricated through thin film hydration technique followed by extrusion. The extruded Dox loaded vesicles achieved through active loading were associated with an optimum size and size distribution, enhanced entrapment within vesicles and improved physicochemical and colloidal stability. The L-PC, DPPC, DSPE-(PEG)2k, and chol based vesicles resulted in the achievement of mild hyperthermia based LCST and drug release. The ETLs-E_(AL)_ showed elevated temperature (42 °C) based instability, phase transition and in vitro drug release. Likewise, MTLs-E_(AL)_ manifested mild hyperthermia (40 °C) based instability, phase transition, in vitro drug release, in vitro cytotoxicity. In addition, an improved cytotoxicity, optimum IC_50_ values and fluorescence microscopy sufficiently provide evidence regarding quick and mild hyperthermia assisted improved cell internalization and tumor targeting. Furthermore, an in vivo healing study corroborated the safety and mild hyperthermia based improved healing of HCC through the developed formulations. Moreover, the requirements associated with passive targeting has also been fulfilled including nanosize ≤ 200 nm, achieved through track-etched 100 nm polycarbonate membrane-based extrusion and stealth effects through PEGylated lipid (DSPE-(PEG)2k). In short, the fabricated liposomes formulations provide enough perspective regarding safe and effective chemotherapy-based treatment approach for hepatocellular carcinoma. The current study could be extended in future by developing multiple stimuli responsive liposomes for active and passive targeting through surface modification and selecting suitable stimuli responsive lipids. In addition, other loading techniques could be explored in the future.

## Figures and Tables

**Table 1:** Formulation composition and physicochemical evaluation of ETLs and MTLs.

Formulation Code	DPPC:DSPE-(PEG)_2k_:L-PC:Chol (µmol%)	EDC (%)		Zeta Size (nm)		Zeta Potential (mV)		PDI	
		CH	AL	NE	E	NE	E	NE	E
ETL1	190:8:0:2	22.56 ± 3.55	28.71 ± 2.01	150.40 ± 2.30	118.20 ±2.81	−13.08 ± 0.12	−13.27 ± 0.04	0.41 ± 0.01	0.24 ± 0.02
ETL2	184:12:0:4	28.80 ± 2.78	44.22 ± 1.70	216.03 ± 5.35	151.70 ±6.45	−22.30 ± 0.14	−22.33 ± 0.20	0.64 ± 0.05	0.33 ± 0.02
ETL3	178:16:0:6	38.33 ± 2.47	51.26 ± 1.85	500.83 ± 9.95	187.13 ±4.15	−22.52 ± 0.14	−22.63 ± 0.31	0.61 ± 0.04	0.43 ± 0.01
MTL1	182:8:8:2	52.06 ± 3.24	79.24 ± 2.16	318.50 ± 2.90	144.63 ±2.43	−28.22 ± 0.39	−29.32 ± 0.41	0.28 ± 0.01	0.21 ± 0.02
MTL2	176:8:12:4	44.22 ± 2.47	58.57 ± 1.23	342.02 ± 3.14	168.01 ±3.08	−29.38 ± 0.41	−30.60 ± 0.51	0.45 ± 0.03	0.29 ± 0.01
MTL3	170:8:16:6	43.50 ± 1.85	61.77 ± 2.16	345.43 ± 3.02	172.76 ±3.15	−31.69 ± 0.13	−32.34 ± 0.15	0.80 ± 0.04	0.24 ± 0.01

*n* = 3 ± SD; CH, conventional hydration technique; AL, Active loading technique; E, extruded; NE, non-extruded.

**Table 2:** Mathematical modeling associated with mechanism of in vitro drug release at different temperatures based on ETLs-E_(AL)_ and MTLs-E_(AL)_.

**Kinetic Modeling Associated with In Vitro Drug Release at 37 °** **C**									
**Sample Code**	**Zero Order**		**First Order**		**Higuchi Model**		**Korsmeyer–Peppas Model**		
	**R^2^**	**K_0_**	**R^2^**	**K_1_**	**R^2^**	**K_H_**	**R^2^**	**K_KP_**	***n***
ETL1-E_(AL)_	0.0141	18.26	0.2820	0.27	0.7862	29.01	0.9921	33.47	0.23
ETL2-E_(AL)_	0.4979	22.19	0.7255	0.36	0.8844	34.29	0.9040	36.36	0.40
ETL3-E_(AL)_	0.0044	21.69	0.3598	0.36	0.7794	34.58	0.9777	39.85	0.23
MTL1-E_(AL)_	0.1406	18.06	0.1561	0.27	0.7160	28.97	0.9832	33.89	0.20
MTL2-E_(AL)_	0.0233	18.25	0.2981	0.27	0.7924	29.05	0.9873	33.46	0.23
MTL3-E_(AL)_	0.2676	19.19	0.4930	0.29	0.8704	31.00	0.9779	32.82	0.30
**Kinetic Modeling Associated with In Vitro Drug Release at 40 °** **C**									
**Sample Code**	**Zero Order**		**First Order**		**Higuchi Model**		**Korsmeyer–Peppas Model**		
	**R^2^**	**K_0_**	**R^2^**	**K_1_**	**R^2^**	**K_H_**	**R^2^**	**K_KP_**	***n***
ETL1-E_(AL)_	0.6964	20.36	0.2931	0.33	0.4082	33.38	0.9930	40.86	0.08
ETL2-E_(AL)_	0.5091	20.60	0.1081	0.34	0.5310	33.54	0.9988	40.49	0.12
ETL3-E_(AL)_	0.7285	27.32	0.0287	0.67	0.3997	44.92	0.9974	55.05	0.08
MTL1-E_(AL)_	0.9479	107.48	0.9666	2.20	0.9763	93.54	0.9989	100.15	0.38
MTL2-E_(AL)_	0.8078	75.46	0.9924	1.86	0.9984	81.14	0.9988	81.12	0.44
MTL3-E_(AL)_	0.7906	78.47	0.9907	2.03	0.9893	84.50	0.9896	84.46	0.44
**Kinetic Modeling Associated with In Vitro Drug Release at 42 °** **C**									
**Sample** **Code**	**Zero Order**		**First Order**		**Higuchi Model**		**Korsmeyer–Peppas Model**		
	**R^2^**	**K_0_**	**R^2^**	**K_1_**	**R^2^**	**K_H_**	**R^2^**	**K_KP_**	***n***
ETL1-E_(AL)_	0.9343	103.48	0.9052	1.97	0.9230	89.42	0.9800	98.11	0.39
ETL2-E_(AL)_	0.9352	97.18	0.9477	1.81	0.9536	84.43	0.9778	90.82	0.36
ETL3-E_(AL)_	0.9001	109.64	0.9425	2.36	0.9712	96.08	0.9798	100.17	0.43
MTL1-E_(AL)_	0.9589	99.62	0.9419	1.99	0.9637	87.49	0.9688	90.36	0.42
MTL2-E_(AL)_	0.8987	96.42	0.9567	1.85	0.9694	84.50	0.9780	88.08	0.44
MTL3-E_(AL)_	0.9079	105.79	0.9755	2.23	0.9874	92.82	0.9948	96.48	0.40

R^2^, regression co-efficien; K_0_, zero order release constant; K_1_, first order release constant; K_H_, Higuchi model release constant; K_KP_, Korsmeyer–Peppas model release constant; *n*, release exponent.

**Table 3:** Comparison of IC_50_ values of Dox solution and MTL1-E_(AL)_ at different temperatures and time intervals.

HepG2 Cell Line				MCF-7 Cell Line		
Sample Code	At Normothermia (37.2 °C)		At Mild Hyperthermia (40.2 °C) after 1 h	At Normothermia (37.2 °C)		At Mild Hyperthermia (40.2 °C) after 1 h
	after 24 h	after 48 h		after 24 h	after 48 h	
Dox solution	5.60	4.65	14.04	5.76	5.07	14.11
MTL1-E_(AL)_	6.84	3.71	3.31	7.49	4.28	3.09

## Data Availability

The data presented in this study are openly available.

## Supplementary Materials

The following is available online at https://www.mdpi.com/article/10.3390/pharmaceutics13081310/s1, Figure S1: Effect of extrusion on the size of developed ETLs and MTLs. Before extrusion as non-extruded ETLs (ETL1-NE, ETL2-NE, and ETL3-NE) and non-extruded MTLs (MTL1-NE, MTL2-NE, and MTL3-NE). After extrusion as extruded ETLs (ETL1-E, ETL2-E, and ETL3-E) and extruded MTLs (MTL1-E, MTL2-E, and MTL3-E).

## References

[1] Horsman M.R., Vaupel P. (2016). Pathophysiological basis for the formation of the tumor microenvironment. Front. Oncol..

[2] Tsoulfas G. (2019). Hepatocellular carcinoma and metabolic syndrome: The times are changing and so should we. World J. Gastroenterol..

[3] Sayiner M., Golabi P., Younossi Z.M. (2019). Disease burden of hepatocellular carcinoma: A global perspective. Dig. Dis. Sci..

[4] Sosa J.A., Bowman H.M., Tielsch J.M., Powe N.R., Gordon T.A., Udelsman R. (1998). The importance of surgeon experience for clinical and economic outcomes from thyroidectomy. Ann. Surg..

[5] Singhal S., Nie S., Wang M.D. (2010). Nanotechnology applications in surgical oncology. Ann. Rev. Med..

[6] Nie S., Xing Y., Kim G.J., Simons J.W. (2007). Nanotechnology applications in cancer. Ann. Rev. Biomed. Eng..

[7] Misra R., Acharya S., Sahoo S.K. (2010). Cancer nanotechnology: Application of nanotechnology in cancer therapy. Drug Discov. Today.

[8] Deamer D.W. (2010). From “Banghasomes” to liposomes: A memoir of Alec Bangham, 1921–2010. FASEB J..

[9] Chellappan D.K., Ng Z.Y., Wong J.-Y., Hsu A., Wark P., Hansbro N., Taylor J., Panneerselvam J., Madheswaran T., Gupta G. (2018). Immunological axis of curcumin-loaded vesicular drug delivery systems. Future Med. Chem..

[10] Tsermentseli S.K., Kontogiannopoulos K.N., Papageorgiou V.P., Assimopoulou A.N. (2018). Comparative study of PEGylated and conventional liposomes as carriers for shikonin. Fluids.

[11] Tagami T., Foltz W.D., Ernsting M.J., Lee C.M., Tannock I.F., May J.P., Li S.-D. (2011). MRI monitoring of intratumoral drug delivery and prediction of the therapeutic effect with a multifunctional thermosensitive liposome. Biomaterials.

[12] Rahim M.A., Jan N., Khan S., Shah H., Madni A., Khan A., Jabar A., Khan S., Elhissi A., Hussain Z. (2021). Recent Advancements in Stimuli Responsive Drug Delivery Platforms for Active and Passive Cancer Targeting. Cancers.

[13] Pattni B.S., Chupin V.V., Torchilin V.P. (2015). New developments in liposomal drug delivery. Chem. Rev..

[14] Cagel M., Grotz E., Bernabeu E., Moretton M.A., Chiappetta D.A. (2017). Doxorubicin: Nanotechnological overviews from bench to bedside. Drug Discov. Today.

[15] Aminsharifi A., Brousell S.C., Chang A., León J., Inman B.A. (2018). Heat-targeted drug delivery: A promising approach for organsparing treatment of bladder cancer. THERMODOX^®^. Arch. Espan. Urol..

[16] Lyon P.C., Griffiths L.F., Lee J., Chung D., Carlisle R., Wu F., Middleton M.R., Gleeson F.V., Coussios C.C. (2017). Clinical trial protocol for TARDOX: A phase I study to investigate the feasibility of targeted release of lyso-thermosensitive liposomal doxorubicin (ThermoDox^®^) using focused ultrasound in patients with liver tumours. J. Ther. Ultrasound.

[17] Lu T., Lokerse W.J.M., Seynhaeve A.L.B., Koning G.A., ten Hagen T.L.M. (2015). Formulation and optimization of idarubicin thermosensitive liposomes provides ultrafast triggered release at mild hyperthermia and improves tumor response. J. Control. Rel..

[18] Kim M.S., Lee D.-W., Park K., Park S.-J., Choi E.-J., Park E.S., Kim H.R. (2014). Temperature-triggered tumor-specific delivery of anticancer agents by cRGD-conjugated thermosensitive liposomes. Coll. Surf. B Bioint..

[19] Abraham S.A., Waterhouse D.N., Mayer L.D., Cullis P.R., Madden T.D., Bally M.B. (2005). The liposomal formulation of doxorubicin. Methods in Enzymology.

[20] de Matos M.B.C., Beztsinna N., Heyder C., Fens M.H.A.M., Mastrobattista E., Schiffelers R.M., Leneweit G., Kok R.J. (2018). Thermosensitive liposomes for triggered release of cytotoxic proteins. Eur. J. Pharm. Biopharm..

[21] Liang Y., Fu X., Du C., Xia H., Lai Y., Sun Y. (2020). Enzyme/pH-triggered anticancer drug delivery of chondroitin sulfate modified doxorubicin nanocrystal. Artif. Cells Nanomed. Biotech..

[22] Sadeghi N., Kok R.J., Bos C., Zandvliet M., Geerts W.J., Storm G., Moonen C.T., Lammers T., Deckers R. (2019). Hyperthermia-triggered release of hypoxic cell radiosensitizers from temperature-sensitive liposomes improves radiotherapy efficacy in vitro. Nanotechnology.

[23] Li S., Yin G., Pu X., Huang Z., Liao X., Chen X. (2019). A novel tumor-targeted thermosensitive liposomal cerasome used for thermally controlled drug release. Int. J. Pharm..

[24] Gao W., Li L., Zhang X., Luo L., He Y., Cong C., Gao D. (2020). Nanomagnetic liposome-encapsulated parthenolide and indocyanine green for targeting and chemo-photothermal antitumor therapy. Nanomedicine.

[25] Mady M.M., Shafaa M.W., Abbase E.R., Fahium A.H. (2012). Interaction of Doxorubicin and Dipalmitoylphosphatidylcholine Liposomes. Cell Biochem. Biophys..

[26] Needham D., Anyarambhatla G., Kong G., Dewhirst M.W. (2000). A new temperature-sensitive liposome for use with mild hyperthermia: Characterization and testing in a human tumor xenograft model. Cancer Res..

[27] Shen J., Burgess D.J. (2013). In vitro dissolution testing strategies for nanoparticulate drug delivery systems: Recent developments and challenges. Drug Deliv. Transl. Res..

[28] Lu T., ten Hagen T.L. (2020). A novel kinetic model to describe the ultra-fast triggered release of thermosensitive liposomal drug delivery systems. J. Control. Rel..

[29] Christensen E., Henriksen J.R., Jørgensen J.T., Amitay Y., Shmeeda H., Gabizon A.A., Kjær A., Andresen T.L., Hansen A.E. (2018). Folate receptor targeting of radiolabeled liposomes reduces intratumoral liposome accumulation in human KB carcinoma xenografts. Int. J. Nanomed..

[30] Jain A., Jain S.K. (2016). In vitro release kinetics model fitting of liposomes: An insight. Chem. Phys. Lipids.

[31] Fatimah A.A., Omaima M. (2016). Histopathological and biochemical studies in male Wistar albino rats injected with diethylnitrosamine and treated with Camel’s milk and Curcuma longa. Egypt. J. Chem. Environ. Health.

[32] Abo Mansour H., El-Batsh M., Badawy N., Mehanna E., Mesbah N., Abo-Elmatty D. (2020). Effect of co-treatment with doxorubicin and verapamil loaded into chitosan nanoparticles on diethylnitrosamine-induced hepatocellular carcinoma in mice. Human Exp. Toxicol..

[33] Pittala S., Krelin Y., Shoshan-Barmatz V. (2018). Targeting liver cancer and associated pathologies in mice with a mitochondrial VDAC1-based peptide. Neoplasia.

[34] Madni A., Rahim M.A., Mahmood M.A., Jabar A., Rehman M., Shah H., Khan A., Tahir N., Shah A. (2018). Enhancement of dissolution and skin permeability of pentazocine by proniosomes and niosomal gel. AAPS Pharm. Sci. Tech..

[35] Khan S., Madni A., Rahim M.A., Shah H., Jabar A., Khan M.M., Khan A., Jan N., Mahmood M.A. (2021). Enhanced in vitro release and Permeability of Glibenclamide by Proliposomes: Development, Characterization and Histopathological Evaluation. J. Drug Deliv. Sci. Technol..

[36] Kneidl B., Peller M., Winter G., Lindner L.H., Hossann M. (2014). Thermosensitive liposomal drug delivery systems: State of the art review. Int. J. Nanomed..

[37] Dorjsuren B., Chaurasiya B., Ye Z., Liu Y., Li W., Wang C., Shi D., Evans C.E., Webster T.J., Shen Y. (2020). Cetuximab-Coated Thermo-Sensitive Liposomes Loaded with Magnetic Nanoparticles and Doxorubicin for Targeted EGFR-Expressing Breast Cancer Combined Therapy. Int. J. Nanomed..

[38] Hsu H.-L., Chen J.-P. (2017). Preparation of thermosensitive magnetic liposome encapsulated recombinant tissue plasminogen activator for targeted thrombolysis. J. Mag. Mag. Mat..

[39] Ong S.G.M., Chitneni M., Lee K.S., Ming L.C., Yuen K.H. (2016). Evaluation of extrusion technique for nanosizing liposomes. Pharmaceutics.

[40] Hossann M., Wang T., Wiggenhorn M., Schmidt R., Zengerle A., Winter G., Eibl H., Peller M., Reiser M., Issels R.D. (2010). Size of thermosensitive liposomes influences content release. J. Control. Rel..

[41] Bhatia N.M., Gaikwad V.L., Mane R.V., Dhavale R.P., Bhatia M.S. (2018). Quantitative structure–property relationship modeling for the prediction of hydrophilic drug entrapment in liposomes for lung targeted delivery. New J. Chem..

[42] Chiu G.N.C., Abraham S.A., Ickenstein L.M., Ng R., Karlsson G., Edwards K., Wasan E.K., Bally M.B. (2005). Encapsulation of doxorubicin into thermosensitive liposomes via complexation with the transition metal manganese. J. Control. Rel..

[43] Shi D., Mi G., Shen Y., Webster T.J. (2019). Glioma-targeted dual functionalized thermosensitive Ferri-liposomes for drug delivery through an in vitro blood–brain barrier. Nanoscale.

[44] Almeida J.A., Morán M.C., Infante M.R., Pais A. (2010). Interaction of arginine-based cationic surfactants with lipid membranes. An experimental and molecular simulation study. ARKIVOC.

[45] Tahir N., Madni A., Kashif P.M., Rehman M., Raza A., Khan M.I., Rahim M.A., Jabar A. (2017). Formulation and compatibility assessment of PLGA/lecithin based lipid-polymer hybrid nanoparticles containing doxorubicin. Acta Pol. Pharm-Drug Res..

[46] Danmaigoro A., Selvarajah G.T., Noor M.H.M., Mahmud R., Zakaria M., Bakar Z.A. (2017). Development of cockleshell (Anadara granosa) derived CaCO3 nanoparticle for doxorubicin delivery. J. Comput. Theor. Nanosci..

[47] Ghosh S., Lalani R., Maiti K., Banerjee S., Patel V., Bhowmick S., Misra A. (2020). Optimization and efficacy study of synergistic vincristine coloaded liposomal doxorubicin against breast and lung cancer. Nanomedicine.

[48] Huang X., Li M., Bruni R., Messa P., Cellesi F. (2017). The effect of thermosensitive liposomal formulations on loading and release of high molecular weight biomolecules. Int. J. Pharm..

[49] Affram K., Udofot O., Singh M., Krishnan S., Reams R., Rosenberg J., Agyare E. (2017). Smart thermosensitive liposomes for effective solid tumor therapy and in vivo imaging. PLoS ONE.

[50] Ma M., Lei M., Tan X., Tan F., Li N. (2016). Theranostic liposomes containing conjugated polymer dots and doxorubicin for bio-imaging and targeted therapeutic delivery. RSC Adv..

[51] Huff T.B., Tong L., Zhao Y., Hansen M.N., Cheng J.-X., Wei A. (2007). Hyperthermic effects of gold nanorods on tumor cells. Future Med..

[52] Dar K., Ali S., Ejaz M., Nasreen S., Ashraf N., Gillani S., Shafi N., Safeer S., Khan M., Andleeb S. (2019). In vivo induction of hepatocellular carcinoma by diethylnitrosoamine and pharmacological intervention in Balb C mice using Bergenia ciliata extracts. Braz. J. Biol..

