# Supplementary material for: Mild Hyperthermia Responsive Liposomes for Enhanced In Vitro and In Vivo Anticancer Efficacy of Doxorubicin against Hepatocellular Carcinoma
Source: Pharmaceutics. 2021 Aug 21;13(8):1310. doi: 10.3390/pharmaceutics13081310 (PMC8400916; doi:10.3390/pharmaceutics13081310)

Size Distribution Report by Intensity

v2.2

Sample Details

**Sample Name:**	ETL1 (E)
**SOP Name:**	mansettings.nano
**General Notes:**	

**File Name:**	Rsults.dts	**Dispersant Name:**	PBS
**Record Number:**	4949	**Dispersant RI:**	1.330
**Material RI:**	1.59	**Viscosity (cP):**	0.8872
**Material Absorbtion:**	0.010	**Measurement Date and Time:**	Tuesday, September 03, 2019...

System

**Temperature (°C):**	25.0	**Duration Used (s):**	100
**Count Rate (kcps):**	99.2	**Measurement Position (mm):**	4.65
**Cell Description:**	Disposable sizing cuvette	**Attenuator:**	11

Results

			Size (d.nm):	% Intensity:	St Dev (d.n...
**Z-Average (d.nm):**	115.1	**Peak 1:**	200.8	86.1	69.21
**PdI:**	0.266	**Peak 2:**	26.77	11.9	4.898
**Intercept:**	0.807	**Peak 3:**	0.000	0.0	0.000
**Result quality :**	**Good**				

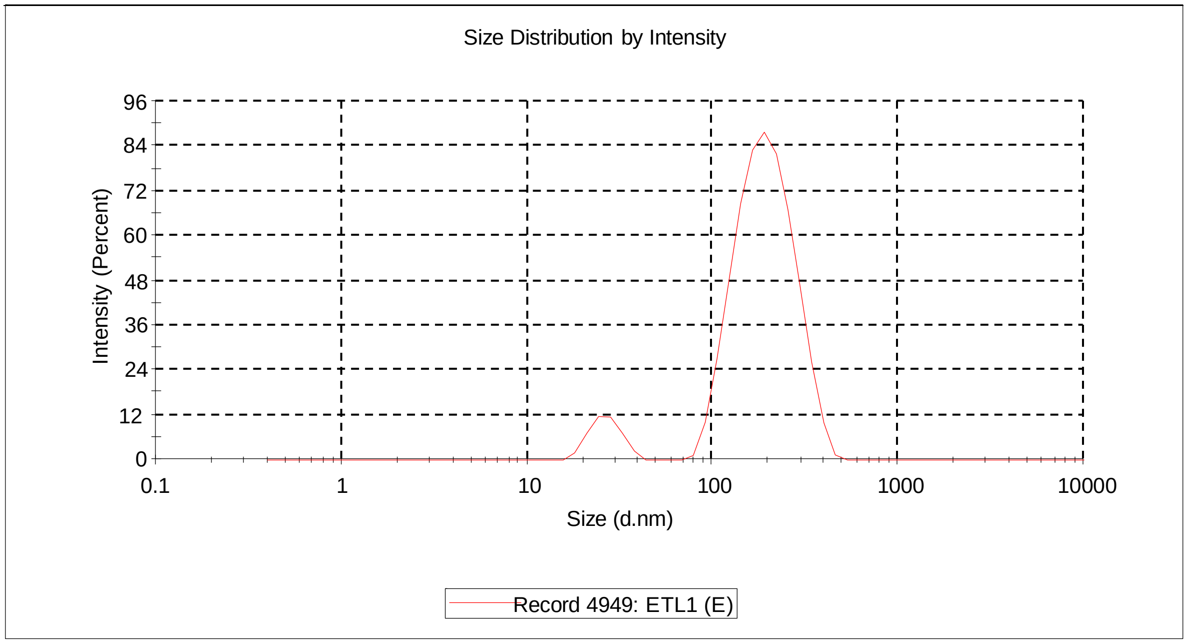

Size Distribution Report by Intensity

v2.2

Sample Details

**Sample Name:**	ETL-1 (NE)
**SOP Name:**	mansettings.nano
**General Notes:**	

**File Name:**	Rsults.dts	**Dispersant Name:**	PBS
**Record Number:**	4950	**Dispersant RI:**	1.330
**Material RI:**	1.59	**Viscosity (cP):**	0.8872
**Material Absorbtion:**	0.010	**Measurement Date and Time:**	Tuesday, September 03, 2019...

System

**Temperature (°C):**	25.0	**Duration Used (s):**	100
**Count Rate (kcps):**	189.1	**Measurement Position (mm):**	4.65
**Cell Description:**	Disposable sizing cuvette	**Attenuator:**	11

Results

			Size (d.nm):	% Intensity:	St Dev (d.n...
**Z-Average (d.nm):**	152.7	**Peak 1:**	214.5	78.9	127.2
**PdI:**	0.422	**Peak 2:**	42.65	18.0	10.85
**Intercept:**	0.882	**Peak 3:**	4826	1.1	713.1

Result quality : Good

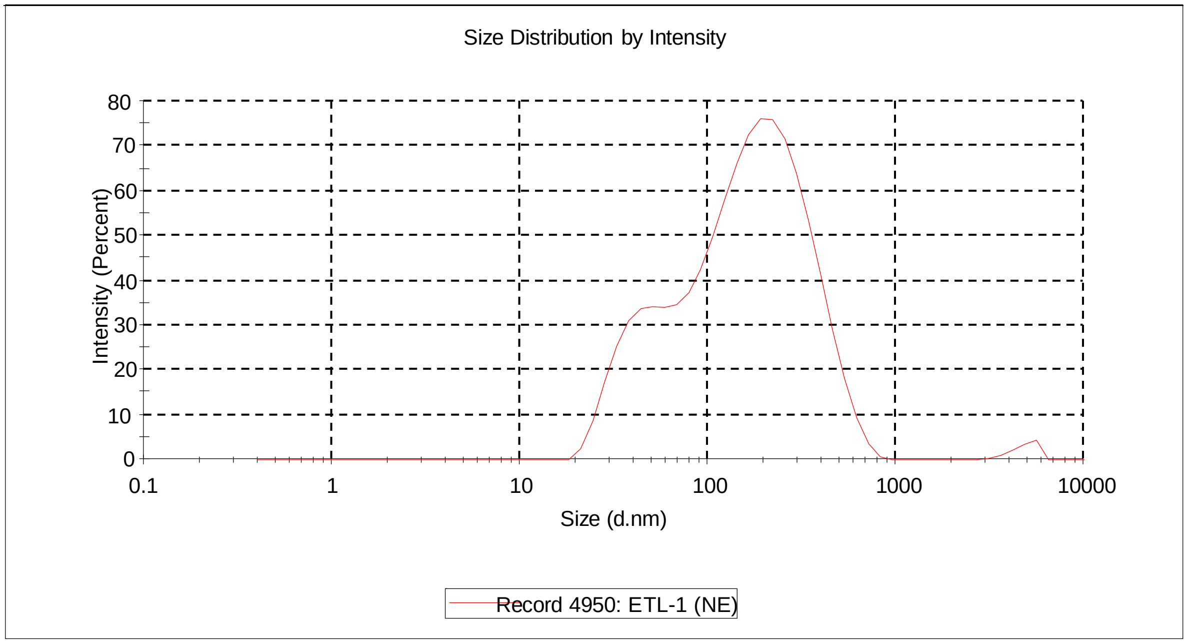

Size Distribution Report by Intensity

v2.2

Malvern

Sample Details

Sample Name: ETL-2 (E)

SOP Name: mansetting.nano

General Notes:

File Name: Rsults.dts

Record Number: 5346

Material RI: 1.59

Material Absorbtion:

Dispersant Name: PBS

Dispersant RI: 1.330

Viscosity (cP): 0.8872

Measurement Date and Time: Monday, October 07, 2019 2:4...

System

Temperature (°C): 25.0

Count Rate (kcps): 210.6

Cell Description: Disposable sizing cuvette

Duration Used(s): 70

Measurement Position (mm): 4.65

Attenuator: 10

Results

Z-Average (d.nm): 151.8

PdI: 0.352

Intercept: 0.959

Result quality : Good

	Size (d.nm):	% Intensity:	St Dev (d.n...
Peak 1:	173.8	92.6	148.2
Peak 2:	77.19	6.3	19.22
Peak 3:	2560	1.1	0.000

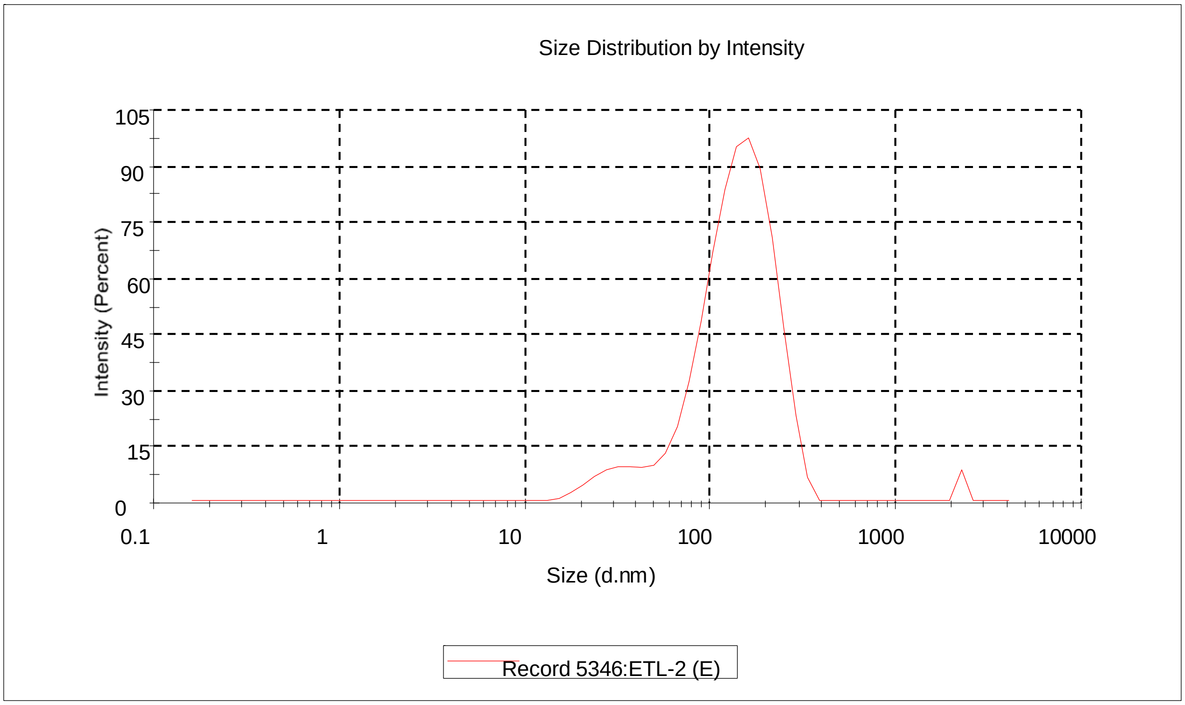

Size Distribution Report by Intensity

v2.2

Sample Details

Sample Name: ETL-2 (NE)

SOP Name: mansettings.nano

General Notes:

**File Name:**	Rsults.dts	**Dispersant Name:**	PBS
**Record Number:**	3078	**Dispersant RI:**	1.330
**Material RI:**	1.59	**Viscosity (cP):**	0.8872
**Material Absorbtion:**	0.010	**Measurement Date and Time:**	Friday, March 15, 2019 11:41:...

System

**Temperature (°C):**	25.0	**Duration Used (s):**	60
**Count Rate (kcps):**	443.4	**Measurement Position (mm):**	4.65
**Cell Description:**	Disposable sizing cuvette	**Attenuator:**	11

Results

			**Size (d.nm):**	**% Intensity:**	**St Dev (d.n...**
**Z-Average (d.nm):**	220.9	**Peak 1:**	377.2	70.1	122.4
**PdI:**	0.615	**Peak 2:**	61.94	26.8	19.70
**Intercept:**	0.871	**Peak 3:**	5471	11.4	243.8

Result quality : **Refer to quality report**

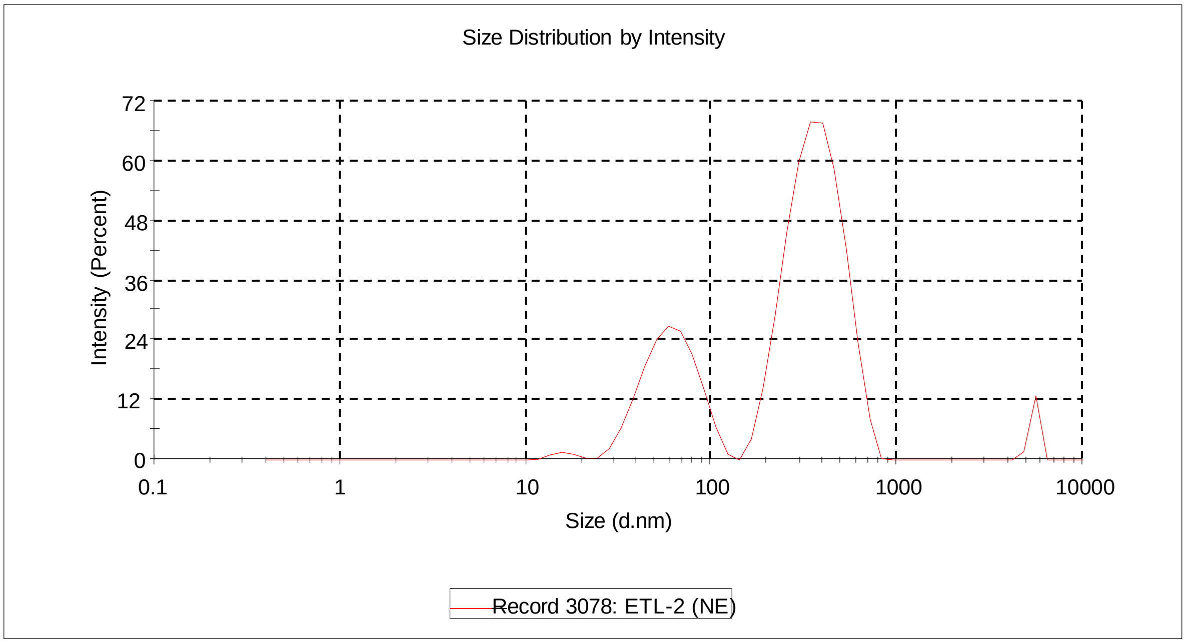

Size Distribution Report by Intensity
v2.2

Sample Details

Sample Name: ETL-3 (E)

SOP Name: mansettings.nano

General Notes:

File Name: Rsults.dts

Record Number: 5347

Dispersant Name: PBS

Material RI: 1.59

Dispersant RI: 1.330

Material Absorbtion: 0.010

Viscosity (cP): 0.8872

Measurement Date and Time: Monday, October 07, 2019

System

Temperature (°C): 25.0

Duration Used (s): 70

Count Rate (kcps): 203.5

Measurement Position (mm): 4.65

Cell Description: Disposable sizing cuvette

Attenuator: 10

Results

		Size (d.nm):	% Intensity:	St Dev (d.n...
**Z-Average (d.nm):** 182.9	**Peak 1:**	152.7	97.0	385.8
**PdI:** 0.425	**Peak 2:**	1798	16.0	717.9
**Intercept:** 0.943	**Peak 3:**	0.000	0.0	0.000

Result quality : Good

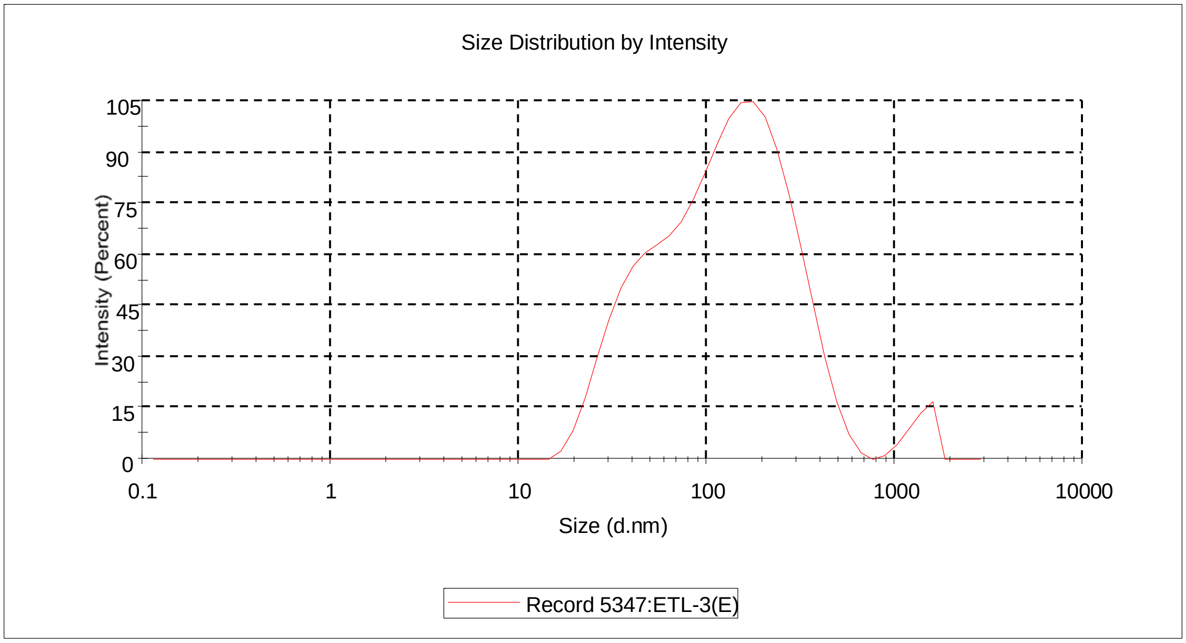

Size Distribution Report by Intensity

v2.2

Malvern

Sample Details

Sample Name: ETL-3 (NE)

SOP Name: mansettings.nano

General Notes:

**File Name:**	Example Results.dts	**Dispersant Name:**	PBS
**Record Number:**	2000	**Dispersant RI:**	1.330
**Material RI:**	1.59	**Viscosity (cP):**	0.8872
**Material Absorbtion:**	0.010	**Measurement Date and Time:**	Tuesday, July 30, 2019 11:19:...

System

**Temperature (°C):**	25.0	**Duration Used (s):**	60
**Count Rate (kcps):**	399.9	**Measurement Position (mm):**	4.65
**Cell Description:**	Disposable sizing cuvette	**Attenuator:**	10

Results

			Size (d.nm):	% Intensity:	St Dev (d.n...
**Z-Average (d.nm):**	510.8	**Peak 1:**	2103	70.6	603.5
**PdI:**	0.617	**Peak 2:**	229.8	61.4	46.57
**Intercept:**	0.930	**Peak 3:**	0.000	0.0	0.000

Result quality : Good

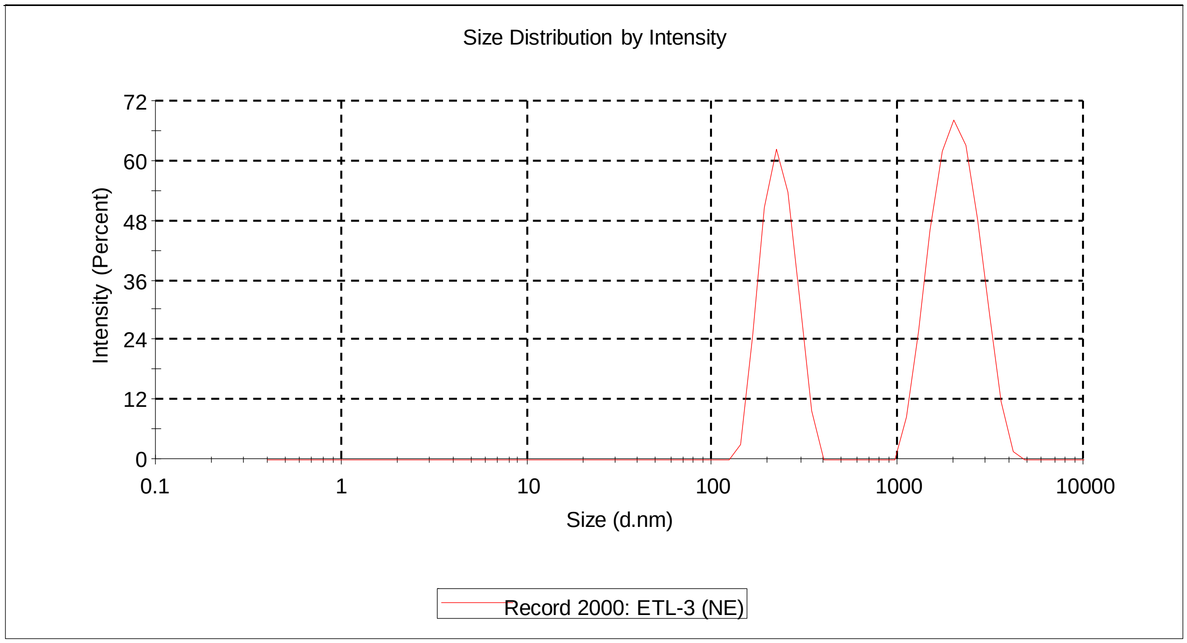

Size Distribution Report by Intensity

v2.2

Sample Details

Sample Name: MTL 1 (E)

SOP Name: mansettings.nano

General Notes:

File Name: Example Results.dts

Record Number: 1989

Material RI: 1.59

Material Absorbtion: 0.010

Dispersant Name: PBS

Dispersant RI: 1.330

Viscosity(cP): 0.8872

Measurement Date and Time: Tuesday, July 30, 2019 10:24.

System

Temperature (°C): 25.0

Count Rate (kcps): 121.7

Cell Description: Disposable sizing cuvette

Duration Used (s): 80

Measurement Position (mm): 4.65

Attenuator: 10

Results

		Size (d.nm):	% Intensity:	St Dev (d.n...
**Z-Average (d.nm):** 147.4	**Peak 1:**	147.4	100.0	120.1
**PdI:** 0.218	**Peak 2:**	0.000	0.0	0.000
**Intercept:** 0.765	**Peak 3:**	0.000	0.0	0.000

Result quality: Good

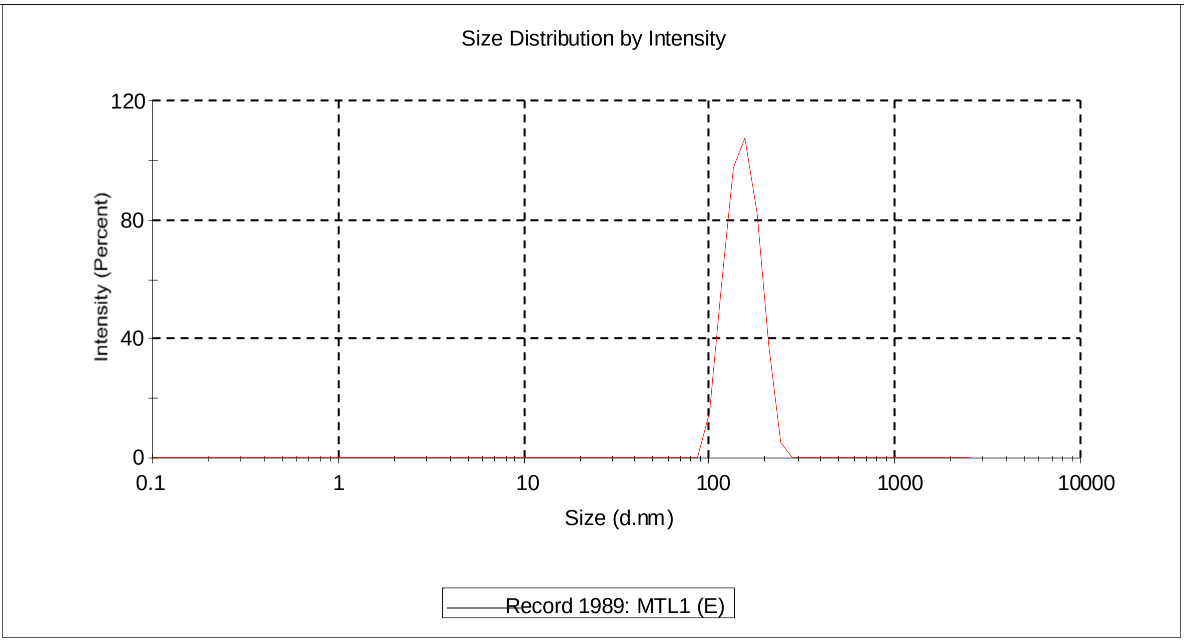

Size Distribution Report by Intensity

v2.2

Malvern

Sample Details

**Sample Name:**	MTL 1 (NE)
**SOP Name:**	particle size.sop
**General Notes:**	Sample(s) was tested according to information provided by customer.

**File Name:**	Rsults.dts	**Dispersant Name:**	PBS
**Record Number:**	5041	**Dispersant RI:**	1.330
**Material RI:**	1.59	**Viscosity (cP):**	0.8872
**Material Absorbtion:**	0.010	**Measurement Date and Time:**	Friday, September 13, 2019 1...

System

**Temperature (°C):**	25.0	**Duration Used (s):**	60
**Count Rate (kcps):**	380.6	**Measurement Position (mm):**	4.65
**Cell Description:**	Disposable sizing cuvette	**Attenuator:**	10

Results

			**Size (d.nm):**	**% Intensity:**	**St Dev (d.n...**
**Z-Average (d.nm):**	318.5	**Peak 1:**	360.2	87.5	126.8
**PdI:**	0.293	**Peak 2:**	5223	9.5	460.3
**Intercept:**	0.893	**Peak 3:**	0.000	0.0	0.000
**Result quality :**	**Good**				

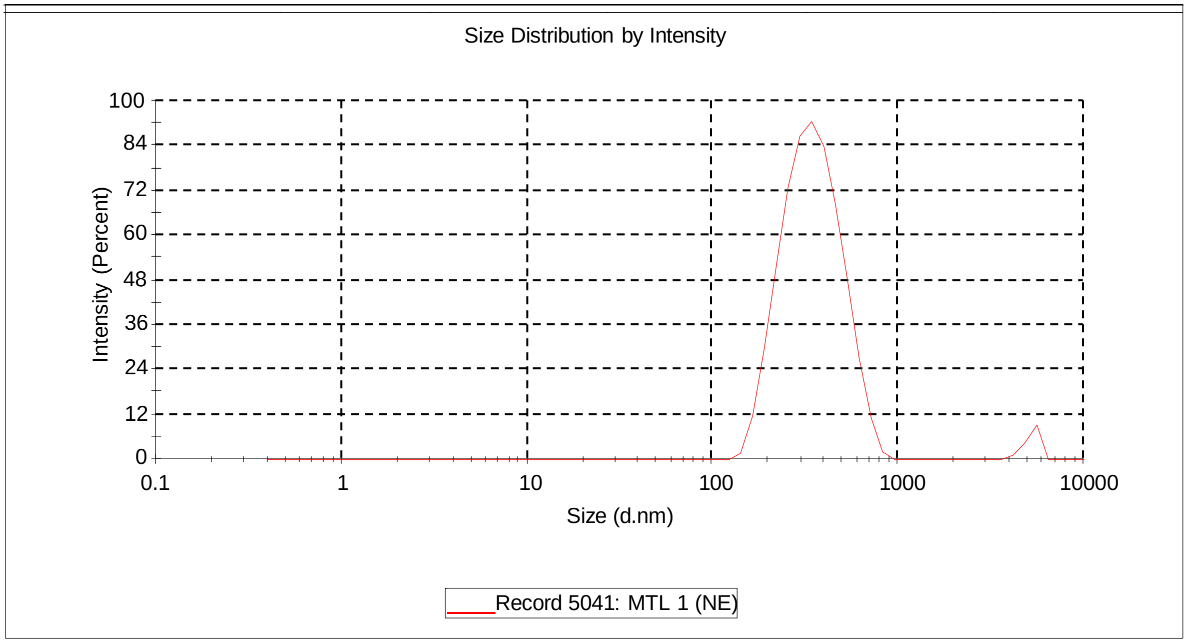

Size Distribution Report by Intensity

v2.2

Malvern

Sample Details

Sample Name: MTL 2 (E)

SOP Name: mansettings.nano

General Notes:

File Name: Rsults.dts

Record Number: 5041

Material RI: 1.59

Material Absorbtion: 0.010

Dispersant Name: PBS

Dispersant RI: 1.330

Viscosity(cP): 0.8872

Measurement Date and Time: Friday, September 13, 2019

System

Temperature (°C): 25.0

Count Rate (kcps): 380.6

Cell Description: Disposable sizing cuvette

Duration Used (s): 60

Measurement Position (mm): 4.65

Attenuator: 10

Results

			Size (d.nm):	% Intensity:	St Dev (d.n....
**Z-Average (d.nm):**	167.5				
**PdI:**	0.293	**Peak 1:**	170.2	70.3	97.5
**Intercept:**	0.893	**Peak 2:**	2223	2.54	126.8
**Result quality:**	Good	**Peak 3:**	0.000	0.0	0.00

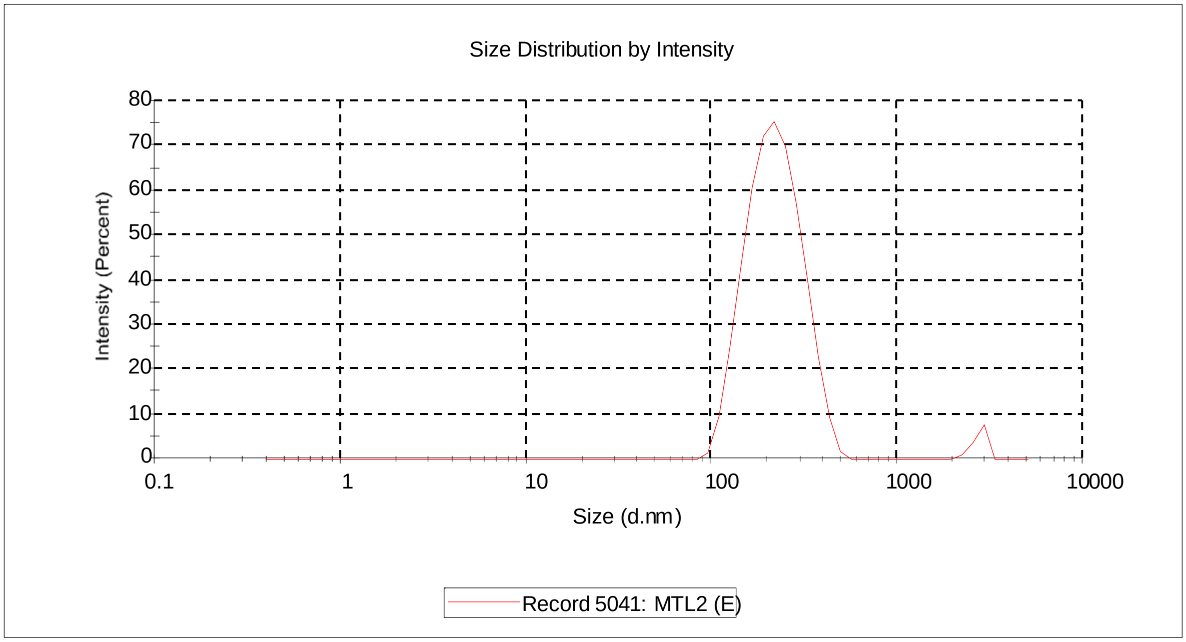

Size Distribution Report by Intensity

v2.2

Malvern

Sample Details

Sample Name: MTL 2 (NE)

SOP Name: mansettings.nano

General Notes:

File Name: Example Results.dts

Record Number: 1358

Material RI: 1.59

Material Absorbtion: 0.010

Dispersant Name: PBS

Dispersant RI: 1.330

Viscosity (cP): 0.8872

Measurement Date and Time: Tuesday, July 30, 2019 10:24.

System

Temperature (°C): 25.0

Count Rate (kcps): 132.2

Cell Description: Disposable sizing cuvette

Duration Used (s): 70

Measurement Position (mm): 0.65

Attenuator: 5

Results

			Size (d.nm):	% Intensity:	St Dev (d.n...
**Z-Average (d.nm):** 341.4		**Peak 1:**	253.8	68.7	149.0
**PdI:** 0.465		**Peak 2:**	53.12	32.2	15.47
**Intercept:** 0.901		**Peak 3:**	3955	19.1	1102

Result quality : Good

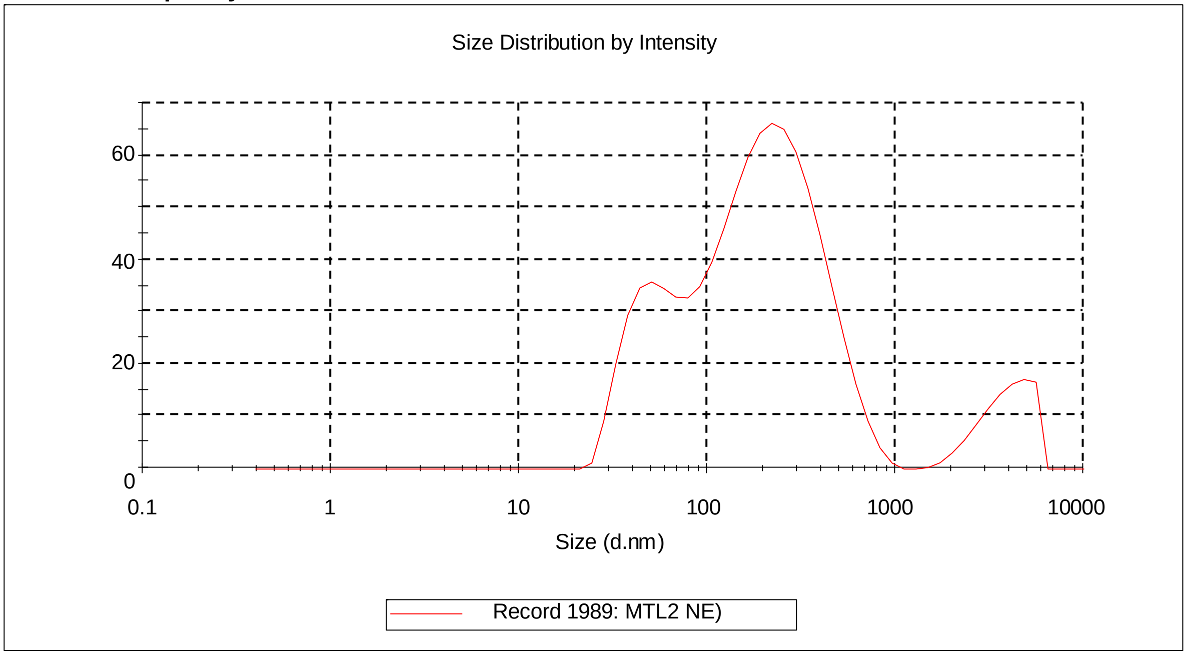

Size Distribution Report by Intensity
v2.2

Malvern

Sample Details

Sample Name: MTL 3 (NE)

SOP Name: mansettings.nano

General Notes:

File Name: Results.dts

Record Number: 1358

Material RI: 1.59

Material Absorbtion: 0.010

Dispersant Name: PBS

Dispersant RI: 1.330

Viscosity (cP): 1.1442

Measurement Date and Time: Tuesday, December 24, 2019...

System

Temperature (°C): 25.0
Count Rate (kcps): 242.0
Cell Description: Disposable sizing cuvette

Duration Used (s): 70
Measurement Position (mm): 0.65
Attenuator: 5

Results

Z-Average (d.nm): 345.9

PdI: 0.830

Intercept: 0.903

Result quality: Good

	Size (d.nm):	% Intensity:	St Dev (d.n...
**Peak 1:**	213.5	49.5	203.2
**Peak 2:**	901.2	35.0	121.4
**Peak 3:**	5332	60.1	9.6

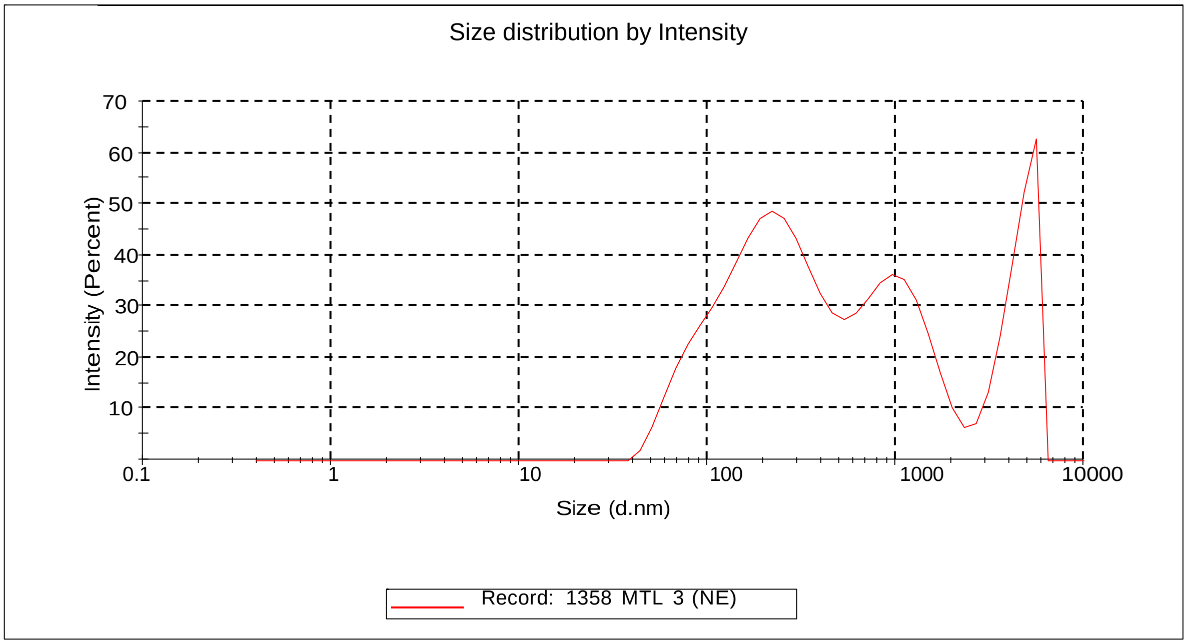

Size Distribution Report by Intensity

v2.2

Malvern

Sample Details

Sample Name: MTL 3 (E)

SOP Name: mansettings.nano

General Notes:

**File Name:**	Rsults.dts	**Dispersant Name:**	PBS
**Record Number:**	5227	**Dispersant RI:**	1.326
**Material RI:**	1.59	**Viscosity (cP):**	0.5476
**Material Absorbtion:**	0.010	**Measurement Date and Time:**	Wednesday, September 25, 2...

System

**Temperature (°C):**	25.0	**Duration Used (s):**	70
**Count Rate (kcps):**	260.9	**Measurement Position (mm):**	4.65
**Cell Description:**	Disposable sizing cuvette	**Attenuator:**	10

Results

			Size (d.nm):	% Intensity:	St Dev (d.n...
**Z-Average (d.nm):**	169.6	**Peak 1:**	169.6	91.0	123.2
**PdI:**	0.251	**Peak 2:**	0.000	0.0	0.000
**Intercept:**	0.897	**Peak 3:**	0.000	0.0	0.000

Result quality : **Good**

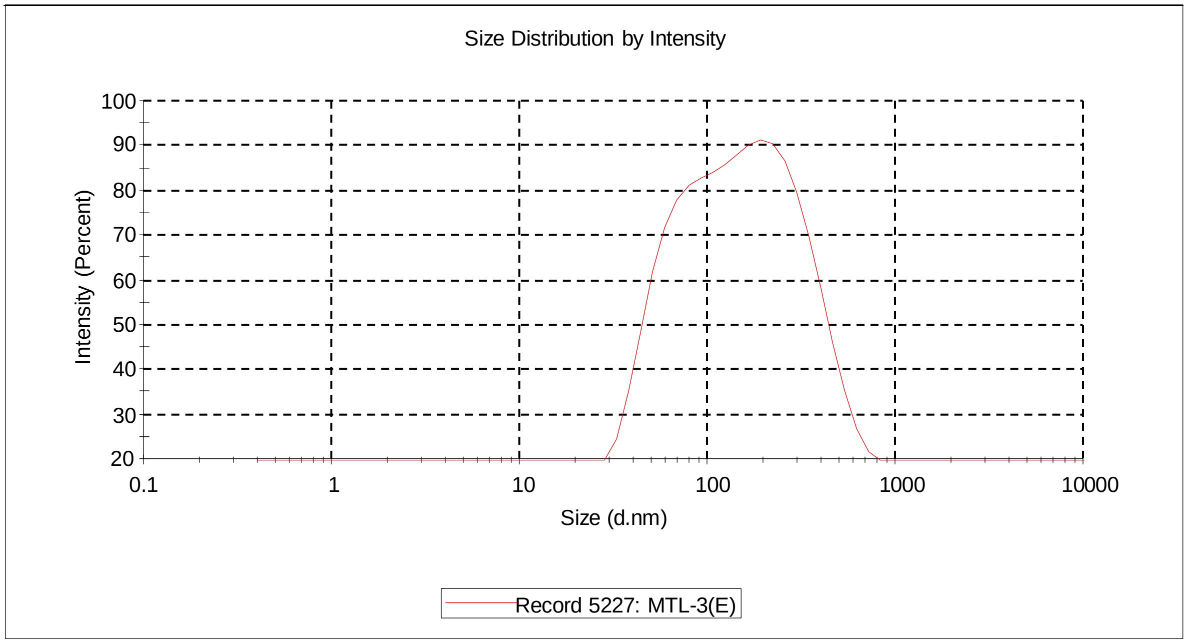

Supplement: Supplementary file 1 [file pharmaceutics-13-01310-s001.zip › pharmaceutics-1305424-supplementary.pdf]
